# Ultrafast Laser Shock Straining in Chiral Chain 2D Materials: Mold Topology-Controlled Anisotropic Deformation

**DOI:** 10.1007/s40820-025-01925-8

**Published:** 2025-11-19

**Authors:** Xingtao Liu, Danilo de Camargo Branco, Licong An, Mingyi Wang, Haoqing Jiang, Ruoxing Wang, Wenzhuo Wu, Gary J. Cheng

**Affiliations:** 1 School of Industrial Engineering, Purdue University, West Lafayette, IN 47906 USA; 2 School of Aeronautics and Astronautics, Purdue University, West Lafayette, IN 47906 USA; 3 School of Materials Engineering, Purdue University, West Lafayette, IN 47906 USA; 4 Birck Nanotechnology Center, Purdue University, West Lafayette, IN 47906 USA

**Keywords:** Tellurene, Laser shock imprinting, Strain engineering, Anisotropic deformation, Chiral chain semiconductor, Dislocation dynamics

## Abstract

Tellurene, a chiral chain semiconductor with a narrow bandgap and exceptional strain sensitivity, emerges as a pivotal material for tailoring electronic and optoelectronic properties via strain engineering. This study elucidates the fundamental mechanisms of ultrafast laser shock imprinting (LSI) in two-dimensional tellurium (Te), establishing a direct relationship between strain field orientation, mold topology, and anisotropic structural evolution. This is the first demonstration of ultrafast LSI on chiral chain Te unveiling orientation-sensitive dislocation networks. By applying controlled strain fields parallel or transverse to Te’s helical chains, we uncover two distinct deformation regimes. Strain aligned parallel to the chain’s direction induces gliding and rotation governed by weak interchain interactions, preserving covalent intrachain bonds and vibrational modes. In contrast, transverse strain drives shear-mediated multimodal deformations—tensile stretching, compression, and bending—resulting in significant lattice distortions and electronic property modulation. We discovered the critical role of mold topology on deformation: sharp-edged gratings generate localized shear forces surpassing those from homogeneous strain fields via smooth CD molds, triggering dislocation tangle formation, lattice reorientation, and inhomogeneous plastic deformation. Asymmetrical strain configurations enable localized structural transformations while retaining single-crystal integrity in adjacent regions—a balance essential for functional device integration. These insights position LSI as a precision tool for nanoscale strain engineering, capable of sculpting 2D material morphologies without compromising crystallinity. By bridging ultrafast mechanics with chiral chain material science, this work advances the design of strain-tunable devices for next-generation electronics and optoelectronics, while establishing a universal framework for manipulating anisotropic 2D systems under extreme strain rates. This work discovered crystallographic orientation-dependent deformation mechanisms in 2D Te, linking parallel strain to chain gliding and transverse strain to shear-driven multimodal distortion. It demonstrates mold geometry as a critical lever for strain localization and dislocation dynamics, with sharp-edged gratings enabling unprecedented control over lattice reorientation. Crucially, the identification of strain field conditions that reconcile severe plastic deformation with single-crystal retention offers a pathway to functional nanostructure fabrication, redefining LSI’s potential in ultrafast strain engineering of chiral chain materials.

**Supplementary Information:**

The online version contains supplementary material available at 10.1007/s40820-025-01925-8.

## Highlights

- Realized ultrafast laser shock imprinting on chiral chain tellurene: Reveals crystallographic orientation-dependent deformation in 2D tellurium via laser shock imprinting.
- Dual deformation regimes: Identifies two distinct strain response modes—parallel strain enables chain gliding and rotation, while transverse strain induces multimodal shear-driven deformations, dramatically altering lattice structure and properties.
- Mold topology enabled strain localization and single-crystal retention—sharp edges generate localized shear, forming dislocations more effectively than smooth molds. Asymmetric strain achieves dense deformation while preserving single-crystal zones, enabling precise optoelectronic nanostructuring.

**Supplementary Information:**

The online version contains supplementary material available at 10.1007/s40820-025-01925-8.

## Graphical abstract

**Figure:**
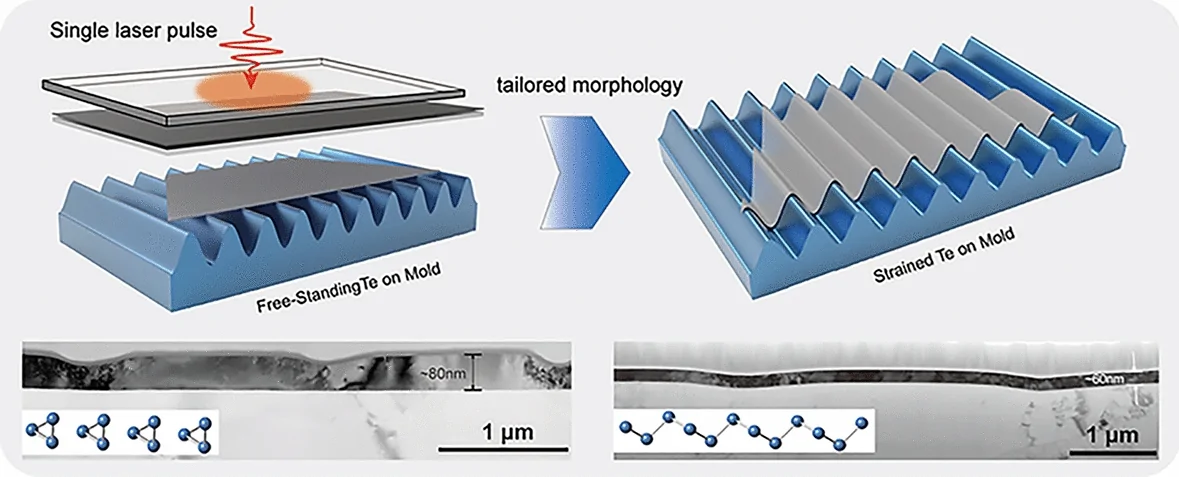

## Introduction

Modulating properties of low-dimensional materials through strain engineering have been studied for various application [1–6]. The exceptional mechanical properties of these materials allow for the introduction of designable strain fields without fracture. Band structure of two-dimensional semiconductors can be tuned efficiently by designing the distribution of strain field. Strain engineering has emerged as a powerful approach to modulate the properties of 2D materials. Nowadays, researchers have investigated different methods to induce strain fields in 2D materials such as lattice mismatch [7] and mechanical buckling techniques [8, 9]. However, controllable, and precise introduction of strain field into 2D devices remains challenging. The laser shock imprinting (LSI) technique offers a high-resolution and precise solution, as the shockwave generated by laser–material interaction can efficiently generate ultrasmooth 3D nanoshaping [10]. By transferring the 2D material onto nanomolds and applying LSI processing, 2D materials can be precisely strained, and the electrical and optical properties can be tailored by designing the nanopatterns. The LSI technique has already been applied in the tailored strain engineering of graphene [11] and transition metal dichloride (TMDC) [12]. The 3D straining induced by LSI technique could opened the bandgap of single-layer graphene and generate nanoscale kink band structures in 2D WSe_2_ crystals.

2D Tellurium (Te), known as tellurene, is a chiral chain semiconducting material with a narrow band gap that has attracted significant attention in recently years due to its unique characteristics [13, 14], including photoconductivity [15–17], high carrier mobility [18, 19] and topological property [18]. The atomic chain of Te is parallel to [0001] direction spirally, each Te atom linked with its two nearest neighbors via covalent bond, and the chains are stacked by weak bonding [19, 20]. Because of the anisotropy of tellurene, the effect of strain on the property change varies along different crystal orientation [21]. Strain engineering in two-dimensional (2D) chiral chain materials, such as tellurene, presents a significant opportunity to manipulate material properties at the nanoscale. Strain engineering is a powerful technique for modifying the intrinsic properties of two-dimensional (2D) materials by inducing lattice deformations, offering significant potential for high-performance devices [20, 21]. Tellurium (Te), an emerging 2D material with attractive characteristics, has been systematically investigated for its strain-engineered anisotropic optical and electrical properties. This capability allows for unprecedented device applications, including flexible strain sensors with a high gauge factor using buckled 2D Te [9]. In the realm of tunable photodetectors, strain engineering has been explored for 2D Te and related materials. β-phase tellurene (β-Te) has been predicted theoretically and fabricated experimentally, showing potential for nanoelectronics and demonstrating that it can sustain large strain [22]. Strain modulation effects have been studied to improve the performance of Te transistors, holding great potential for complementary metal–oxide–semiconductor (CMOS) compatible applications [23]. Additionally, tellurene-based photodetectors have shown bending strain-modulated flexible photodetection in the long-wavelength infrared region, highlighting the impact of strain engineering on their anisotropic properties [24]. However, while LSI has been explored for graphene and TMDCs, the unique chiral chain Te structure poses open questions on how orientation-dependent ultrafast strain affects lattice integrity and device viability. Understanding the deformation mechanisms under ultrafast strain rates remains a critical challenge. How do directional strain fields interact with the unique chiral chain structure of 2D tellurene, and what role do these interactions play in defect formation, dislocation dynamics, and mechanical behavior. Addressing these questions is essential for advancing the precise control of strain in these materials, thereby unlocking their potential for innovative device applications. Investigating these deformation mechanisms through laser shock imprinting on 2D tellurene provides a crucial theoretical foundation for the broader application of strain engineering in chiral chain 2D materials.

This study explores the deformation response of 2D tellurene subjected to ultrafast strain rates, using symmetrical and asymmetrical templates, such as compact disks (CD) and diffraction gratings. The focus lies on the directional dependence of the strain field relative to the [0001] crystallographic direction of tellurium (Te) chains. Parallel strain fields are hypothesized to promote glide and rotation of the chains, while transverse strain fields are expected to introduce complex shear forces, leading to diverse deformation modes, including tensile, compressive, and bending effects. The interplay of these deformation modes with the chiral chain structure of tellurene raises critical questions about the origins and distribution of plastic deformation, particularly the formation of dislocation tangles and their sensitivity to strain field design.

Laser shock imprinting (LSI) emerges as a promising tool for addressing these challenges. By creating controlled and localized strain fields, LSI provides a pathway for precise strain engineering in chiral chain 2D materials. This approach has the potential to redefine how we tailor mechanical, electronic, and optical properties at the nanoscale. The insights gained from studying tellurene’s deformation behavior under ultrafast strain conditions are poised to inform the broader scientific community on strategies for defect control and strain tuning in other 2D materials with anisotropic or chiral structures. These findings carry profound implications for the development of next-generation electronic and optoelectronic devices. By bridging fundamental knowledge gaps in strain engineering and deformation mechanisms, this work lays a foundation for the rational design of strained tellurene-based systems, with far-reaching impact across advanced material and device technologies.

## Experimental Methods

### Materials

The 2D tellurene were synthesized from solution as reported [9, 25]. Then the 2D tellurene flakes were transferred on a 4 μm aluminum foil by Langmuir–Blodgett (LB) method before the LSI processing.

### LSI Processing

**Fig. 1:**
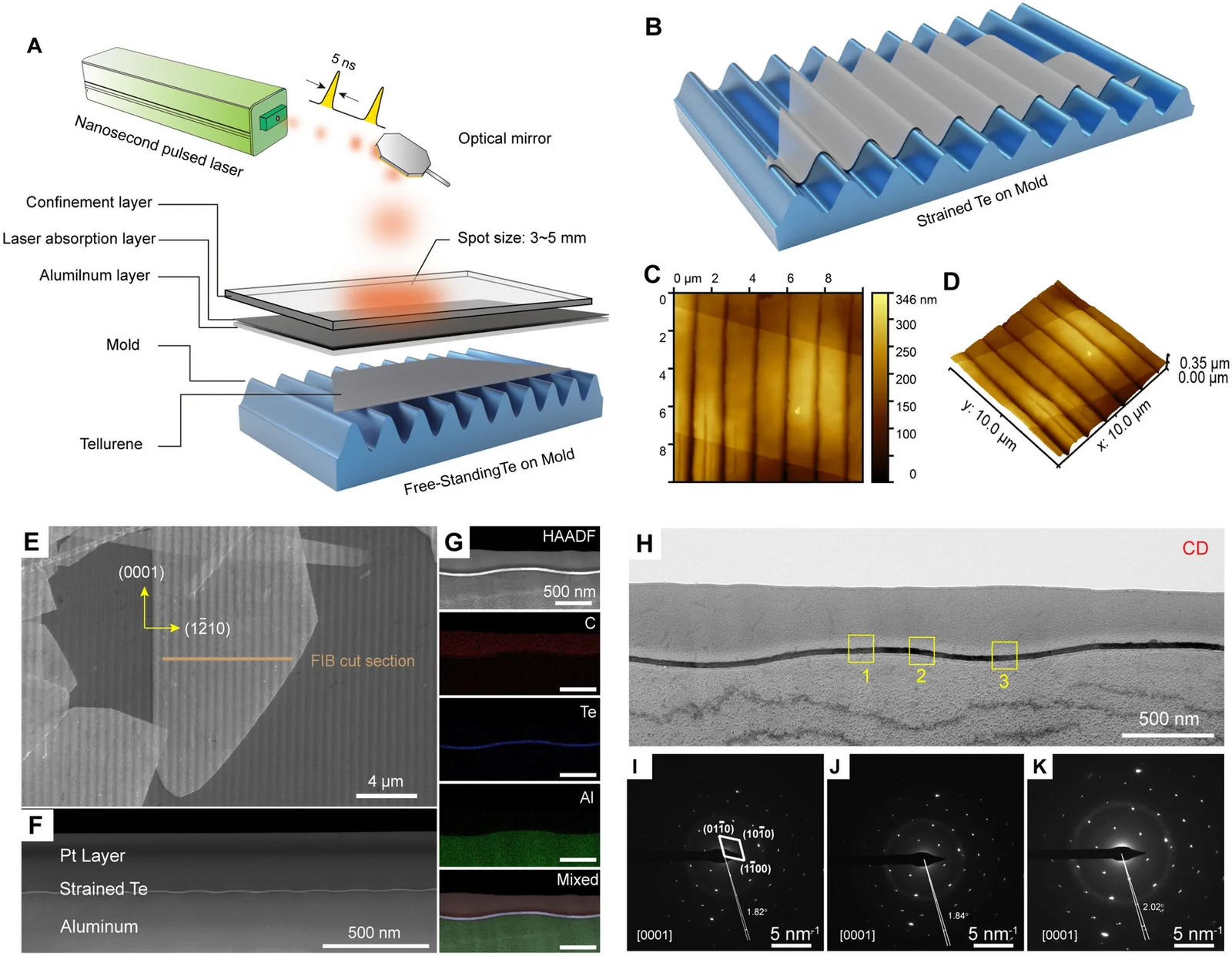
**A-B** Schematic of the LSI processing for the straining of 2D tellurium. **C-D** AFM topography of the strained Te flake on grating mold. **E** SEM images showing the pattern on 2D tellurene and demonstrate the area of TEM sample prep. **F** FIB cross-sectional imaging showing the morphology of the strain 2D tellurene. **G** STEM-EDX mapping showing the distribution of the as-fabricated sample, showing that 2D Te is deformed under the symmetrical deformation on CD mold. **H–K** TEM of the symmetrically strained Te flake on CD mold. € Bright-field TEM image, **I-k** SAD patterns corresponding to the areas in (**H**)

A Continuum Surelite III Q-switch Nd:YAG laser with a wavelength of 1064 nm was used for the straining of 2D tellurene. The pulse energy of the laser beam was 396 mJ and the pulse duration is 5 ns. The laser beam was focused down to 5 mm, therefore, we have a power density was 0.4GW cm^−2^. Aluminum layer is spray coated with graphite as absorption layer and a borosilicate glass is used as a confinement layer. The schematic of the LSI processing is shown in Fig. 1A. The laser beam transmits the glass and interact with the spray-coated graphite (purchased from Asbury Carbons). The high-pressure shock wave generated between the confinement layer and aluminum layer will lead to the deformation of aluminum foil, squeezing the 2D tellurium to the templates. To control the orientation of the induced strain, we used CD and grating templates in this investigation. After transferring the Te flakes onto the Al foil via the Langmuir–Blodgett method, the orientation of both the nanotemplates and the Te flakes can be aligned. This alignment enables effective control over the strain orientation.

### Characterization

Transmission electron microscopy (TEM) analysis and high-resolution transmission electron microscopy (HRTEM) were utilized to characterize the defects formed in the 2D tellurene lattices. A ThermoFisher Scientific 200X transmission electron microscope equipped with a Super-X four-detector energy dispersive X-ray spectrometer (EDX) was used for TEM and HRTEM analysis, which was operated at 200 kV. A double aberration corrected ThermoFisher Scientific Themis Z transmission electron microscope equipped with a high-angle annular dark-field detector (HAADF) was utilized to obtain atomic resolution scanning transmission electron microscopy (STEM) image, which was operated at 300 kV. The TEM samples are prepared by a ThermoFisher Scientific Helios G4 SEM/FIB dual beam system. The ion source of the FIB was operated at 30 kV. Raman spectroscopy and spatial Raman mapping were conducted using a Raman Spectrometer (DXR™3xi Raman Imaging Microscope) with a 50 × objective. Raman maps were collected using 633 nm excitation laser at a spatial resolution of 10 nm. Repeated acquisition using the highest magnification was accumulated to improve the spectrum signal-to-noise ratio. Spectra were calibrated using the 520.5 cm^−1^ line of a silicon wafer.

## Results and Discussion

The schematic of the laser shock imprinting (LSI) processing is illustrated in Fig. 1A. In this experiment, a laser beam with a pulse energy of 396 mJ and a pulse duration of 5 ns was employed. The beam was focused to a diameter of 5 mm, resulting in a power density of 0.1 GW cm^−2^. The experimental setup consisted of an aluminum layer spray-coated with graphite as an absorption layer, and a borosilicate glass serving as a confinement layer. The laser beam transmits through the glass and interacts with the spray-coated graphite, generating a high-pressure shock wave between the confinement layer and the aluminum layer. This resultant force deforms the aluminum foil, which in turn compresses the 2D tellurium against the underlying templates, as depicted in Fig. 1B. A compact disk (CD) was utilized as a nanomold to generate symmetrical strain fields, as shown in Fig. S1A-C, creating a sine wavelike strain pattern.

### Effect of Strain Field Orientation on the Structural Evolution of 2D Te Under LSI

The transmission electron microscopy (TEM) analysis in Fig. 1H illustrates the case where the applied symmetrical strain field is parallel to the [0001] chain direction of 2D Te. TEM samples were prepared across trench patterns to characterize the structural response. Focused ion beam (FIB) cross-sectional images (Fig. 1E, F) reveal the morphological evolution of 2D Te under laser shock imprinting (LSI). The deformed Te layers exhibit a wavy morphology, conforming tightly to the aluminum substrate, suggesting that during LSI processing, the plastic deformation of aluminum facilitated the formation of nanoscale imprints, compressing the Te into these molds. The degree of deformation depends on the laser power density—insufficient power fails to induce significant strain (Fig. S13A, B), while excessive power results in fracturing of the Te flakes (Fig. S13C, D). Elemental STEM-EDX mapping (Fig. 1G) corroborates the FIB cross-sectional imaging, confirming that the deformed Te remains tightly adhered to the aluminum due to its plastic deformation. Bright-field TEM imaging and selected area diffraction (SAD) patterns (Fig. 1I–K) provide further insights into the structural integrity of the deformed Te. While the TEM images reveal no apparent defects within the ~ 20-nm-thick Te flakes, SAD analysis identifies two sets of diffraction patterns rotated by 1.82°, 1.84°, and 2.02°, respectively, across different regions of the sample. This angular shift in diffraction patterns indicates strain-induced lattice rotation, a direct consequence of LSI processing. Notably, despite the imposed strain, the Te flakes retain their single-crystalline nature, demonstrating the ability of Te to accommodate deformation through lattice rotation rather than introducing significant structural defects.

A second experimental scenario investigates the effect of a symmetrical strain field perpendicular to the [0001] chain direction, where Te exhibits distinct deformation characteristics. Along the [0001] direction, Te atoms are covalently bonded, resulting in higher mechanical resistance to deformation under strain applied orthogonal to this axis. The underlying deformation mechanism differs significantly from the parallel case due to the intrinsic anisotropy of Te’s bonding structure. TEM samples were prepared across the trench direction (Fig. S2) to capture microstructural changes in this configuration. Unlike the wavy deformation observed in the parallel strain case, TEM imaging (Fig. S3) reveals the formation of dislocations as the primary accommodation mechanism in 2D Te subjected to ultrafast strain rates. The overall deformation is less pronounced than in the parallel case (Fig. 1E), reflecting the material’s greater resistance to strain along this orientation. Magnified TEM images (Fig. S3B, D, and F) highlight the wavy morphology of dislocation lines, which preferentially align along the [0001] direction. This suggests that, under symmetrical deformation, strain is accommodated through the generation and motion of dislocations rather than large-scale lattice rotation. The corresponding SAD patterns (Fig. S3C, E, and G) indicate that despite the presence of dislocations, the deformed Te flakes maintain their single-crystalline integrity post-LSI processing. However, a notable distinction arises in the SAD pattern comparison: Fig. S3E exhibits a clockwise lattice rotation relative to Fig. S3C, G, signifying localized strain accommodation via lattice distortion rather than uniform deformation. These observations provide critical insights into the strain engineering of Te-based nanostructures, demonstrating the potential for tailored mechanical manipulation of 2D Te properties via controlled strain field orientations.

### 3D Strain Engineering in 2D Tellurium: Anisotropic Deformation Under Ultrafast Laser Shock

In contrast to the homogeneous, symmetrical strain field produced by the CD mold, the blaze grating introduces a periodic sharp-edged topology (Fig. S4) that fundamentally alters the deformation mechanics. The sharp edges generate localized shear forces at regular intervals, which in turn provoke a distinctly non-uniform deformation response in the Te flakes.

#### Periodic Shear-Induced Dislocation Networks

The periodic sharp edges of the blaze grating create zones of high shear stress that locally intensify the mechanical loading. SEM images (Figs. S5 and S6) clearly show that Te flakes directly contacting these sharp edges undergo severe deformation. The cross-sectional view in Fig. S4B confirms that these high-stress regions experience significant plastic deformation. In contrast, the regions of the Te flake that contact the flat portions of the grating are subjected primarily to a laser shock peening process, leading to relatively milder deformation.

**Fig. 2:**
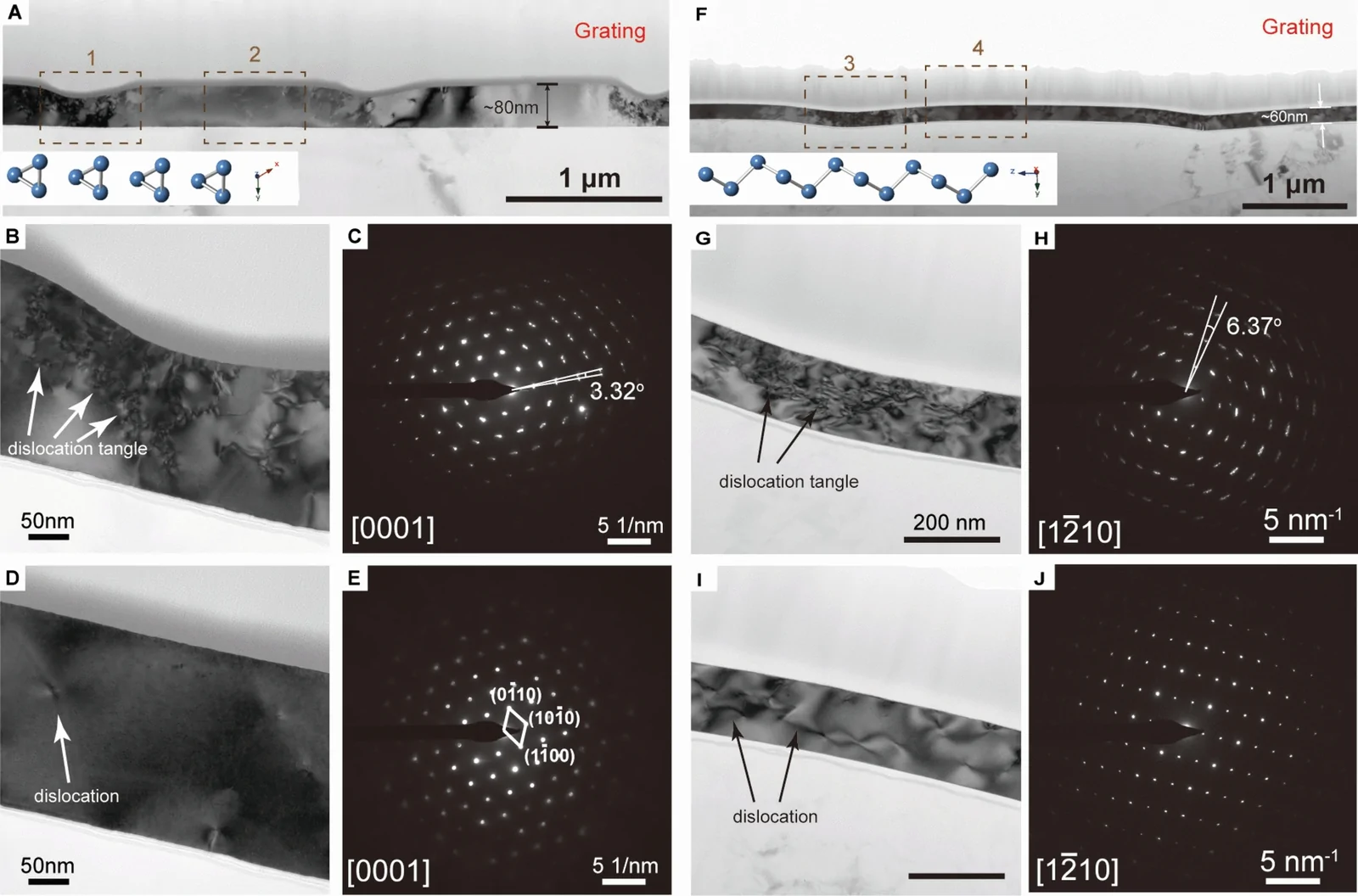
TEM analysis of the asymmetrical strain parallel to the chain direction in Te. **A** Bright-field TEM image showing the morphology of Te flake after LSI on grating mold. **B-C** Magnified image of area 1 and corresponding SAD pattern. **D-E** Magnified image of area 2 and corresponding SAD pattern; **F-J** TEM analysis of the strained Te flake whose [0001] direction is perpendicular to grating edges **F.** Bright-field TEM image showing the morphology of Te flake after LSI on grating mold. **G-H** Magnified image of area 5 and corresponding SAD pattern. **I-J** Magnified image of area 4 and corresponding SAD pattern

TEM analysis (Fig. 2) corroborates the observations from the FIB cross sections (Figs. S4 and S5). When the blaze grating’s edge is aligned parallel to the Te chains, bright-field TEM images (Fig. 2A, B, and D) reveal the formation of dislocation tangles in regions of severe deformation. These dislocation tangles are absent in areas exposed to laser shock peening on flat surface, where the Te flake retains a well-ordered, single-crystal structure with low dislocation density.

Magnified views and corresponding SAD patterns (Fig. 2B–E) provide quantitative insight: In the heavily deformed region (area 2), the diffraction pattern indicates a lattice orientation change of approximately 3.32°. This change is markedly larger than the rotation observed under the more uniform strain field generated by the CD mold parallel to the [0001] direction. In contrast, area 1, which experiences only the shock peening effect, remains nearly undeformed and preserves a pristine single-crystal lattice.

#### Helical Chain Deformation

When the sharp edge of the blaze grating is oriented perpendicular to the helical Te chains, the LSI process imposes significant shear forces directly on the chain structure. As demonstrated by the morphologies in Figs. 1C, D and S5, the Te flake adopts a shape that mirrors the grating’s periodic topography along the thickness direction. The corresponding SAD pattern reveals a substantial lattice orientation change of 6.37°, indicative of severe deformation. Importantly, despite the large angular shift, the lattice remains intact, underscoring the robustness of the Te helical chains under ultrafast strain rates. In adjacent flat regions, the lattice structure retains its single-crystalline character with only a low dislocation density (Fig. 2I, J).

Fundamental mechanistic insights indicate that the periodic sharp edges of the blaze grating introduce highly localized shear forces that markedly exceed the homogeneous strain generated by the CD mold. This elevated shear promotes inhomogeneous deformation, resulting in the formation of dislocation tangles and larger lattice orientation changes, as well as more severe plastic deformation when compared to the uniform strain field. The crystallographic orientation plays a critical role: when the grating edge is parallel to the Te chains, the shear forces primarily induce dislocation formation while largely maintaining lattice continuity; in contrast, when the edge is perpendicular to the helical chains, the shear directly distorts the chain architecture, leading to pronounced lattice rotations without catastrophic failure. These observations underscore the significant impact of mold topology and orientation on the deformation behavior of 2D Te, providing valuable insights into optimizing LSI processes to tailor nanostructured morphologies while preserving intrinsic crystal integrity. The experimental findings elucidate that the periodic sharp edges of the blaze grating induce higher localized shear forces than the smooth, homogeneous strain fields of the CD mold. This results in markedly different deformation mechanisms—ranging from lattice rotations to the formation of dislocation tangles—depending on the relative orientation of the grating edge to the Te chains. Such insights are crucial for advancing strain engineering strategies in 2D materials.

### Mechanistic Discussion of Lattice Structural Changes in Strained 2D Te

High-resolution TEM (HRTEM) and high-resolution STEM (HRSTEM) imaging are employed here to decipher the fundamental mechanisms governing the deformation of 2D tellurium (Te) under various strain conditions. By examining the lattice structure at the atomic level, we can understand how different strain fields—imposed through distinct mold configurations—alter the material’s behavior, which is crucial for tailoring its properties for advanced electronic and optoelectronic applications.

**Fig. 3:**
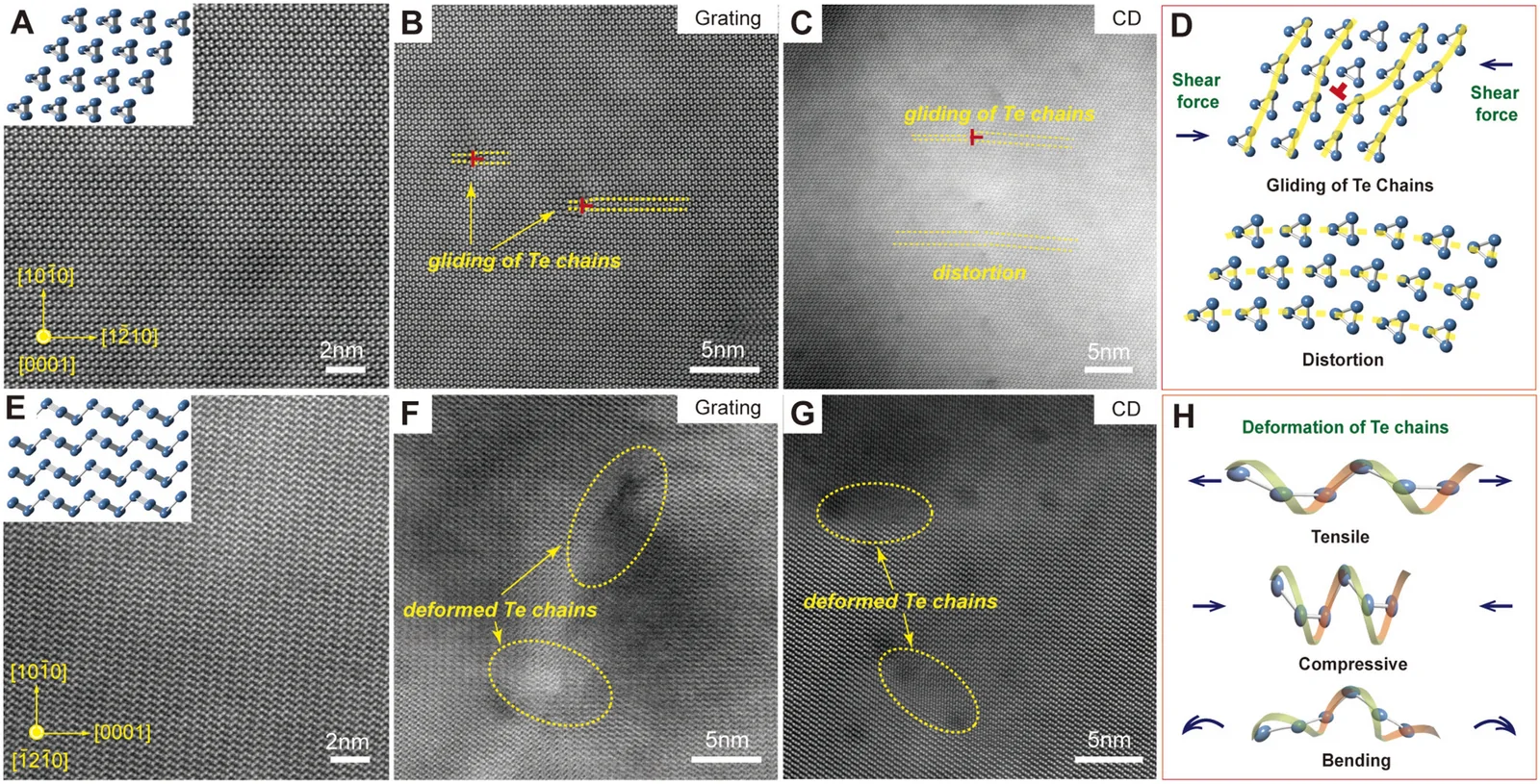
HSTEM images of the strained Te along [0001] zone(A-D) and [$12\overline{1 }0$] zone. **A** and **E** non-deformed area **B** and **F** severely deformed area on grating mold, **C** and **G** deformed area on CD mold, **D** and **H** schematic of the deformation mechanisms of Te flakes under laser shock imprinting

HRSTEM images (Fig. 3A–C) reveal the intricate lattice structures of Te chains in their undeformed state, where a pristine single-crystal structure is maintained with a well-ordered array of chains (as seen in Fig. 3A, E). This pristine structure serves as a baseline, emphasizing the intrinsic structural integrity of the material. However, when the Te flakes are subjected to severe deformation via laser shock imprinting (LSI) on a blaze grating mold, a markedly different mechanism emerges. The imposed high shear stress—localized at the periodic sharp edges of the grating—induces significant chain gliding. This phenomenon manifests as edge dislocation-like structures, which serve as the primary mechanism for accommodating plastic deformation in the material. In contrast, the symmetrical strain field produced by a CD mold not only triggers similar chain gliding but also distorts the interactions between adjacent chains. Such distortions generate additional elastic strain fields within the lattice, further complicating the overall deformation mechanism. These differences are illustrated in Fig. 3D, which outlines the processes of Te chain gliding and distortion, providing valuable insights into the Tellurene's behavior under 3D straining.

Further mechanistic insights are gleaned from HRTEM images (Fig. S7A, C, and E), which demonstrate that the as-grown Te flakes are not atomically flat. This inherent non-uniformity predisposes the material to inhomogeneous deformation under LSI processing, where localized variations in thickness and initial lattice orientation can lead to differential strain responses. Magnified images coupled with fast Fourier transform (FFT) patterns reveal that regions of the flake experience varied rotations and slips of the Te chains. For example, areas 1, 2, and 3 in Fig. 1H show both rotation and slip, suggesting that even minor preexisting imperfections can amplify the complexity of strain accommodation during high-energy processing.

In regions where the Te flake remains flat after LSI, HRSTEM images (Fig. 3E) confirm that the lattice structure retains its original configuration, indicating that not all regions are equally susceptible to deformation. Conversely, in the severely deformed regions on the grating mold (Fig. 3F), the high localized shear forces result in the formation of extensive dislocation tangles. These tangles indicate a significant disruption in the original lattice arrangement, where some helical chains are not only plastically deformed but also partially broken. The formation of these defects—together with the observation of elastic deformations along intact chains (highlighted by yellow dotted areas in Fig. 3F, G)—demonstrates a dual-mode deformation: irreversible plastic changes coexist with reversible elastic responses. This interplay between plasticity and elasticity underscores the complex strain accommodation mechanisms in 2D Te, where the periodic nature of the mold directly influences the spatial distribution of the deformation.

Additional HRTEM images in Fig. S8A, B provide further evidence of strong lattice distortions in symmetrically strained Te flakes. Here, the Te chains are observed to be stretched or compressed along the [0001] direction due to residual stress, which is a direct consequence of the imposed external strain. The appearance of (101̅0) and (101̅1) type dislocations (Fig. S8C–E) confirms that the chains are subject to significant bending, stretching, and compression. These structural changes, documented in the inverse FFT (IFFT) images, reveal that chain deformation is a multifaceted process involving both local atomic rearrangements and more extended dislocation networks.

HRTEM analysis presented in Fig. S9 contrasts the undeformed and severely deformed regions of the Te flake. While the pristine regions exhibit a perfect single-crystal lattice devoid of defects, the zones experiencing intense strain—especially near the sharp edges of the grating—show significant dislocation formation and chain compaction. The rapid, ultrafast strain rate of LSI induces cross-slip of the chains, further complicating the deformation process by creating intricate dislocation tangles. In areas where the strain field is oriented perpendicular to the helical chains (Fig. S10A, B), uniform bending and distortion of the chains are observed, indicating a substantial reorganization of the lattice structure. The retention of crystalline order in certain regions underscores the non-uniform strain distribution across the Te flake, while severely deformed areas exhibit significant lattice distortion. In high-strain regions, the helical chains undergo systematic deformation and bending, deviating substantially from their equilibrium configuration. This structural transformation directly impacts the material’s electronic and mechanical properties by modifying interatomic interactions and charge transport pathways. The coexistence of preserved crystalline domains and highly deformed regions indicates a complex interplay between strain localization and lattice rearrangement. Under extreme deformation, chain breaking, dislocation formation, and extensive lattice disruptions emerge, collectively altering the vibrational and electronic properties of 2D Te. These findings emphasize the crucial role of strain directionality in dictating whether the material predominantly undergoes elastic deformation, dislocation nucleation, or chain scission, thereby offering a pathway for controlled property tuning in electronic and optoelectronic applications.

Comprehensive HRTEM and HRSTEM analyses further elucidate the fundamental deformation mechanisms governing 2D Te’s strain response. The results indicate that the material’s behavior under external stress is governed by an intricate interplay between its intrinsic anisotropic bonding structure, the initial crystal quality, and the topology of the strain engineering mold. High localized shear forces at blaze grating edges facilitate chain gliding and dislocation nucleation, whereas symmetrical strain fields drive both interchain distortion and gliding motions. These observations highlight the necessity of considering both atomic-scale imperfections and macroscopic mold geometry when designing strain engineering processes aimed at optimizing the microstructural integrity and functional properties of 2D Te for advanced device applications.

### Raman Spectroscopy Analysis and Underlying Straining Mechanisms in 2D Te

Raman spectroscopy provides a powerful method to probe lattice vibrations and, by extension, the internal strain state of 2D tellurium (Te) flakes. In our study, Raman mapping under an A^1^ mold was used to investigate the breathing vibration mode in the (101̅0) plane. This mode is highly sensitive to changes in interatomic forces, allowing us to elucidate how applied strains modify the vibrational properties and, by inference, the underlying deformation mechanisms.

**Fig. 4:**
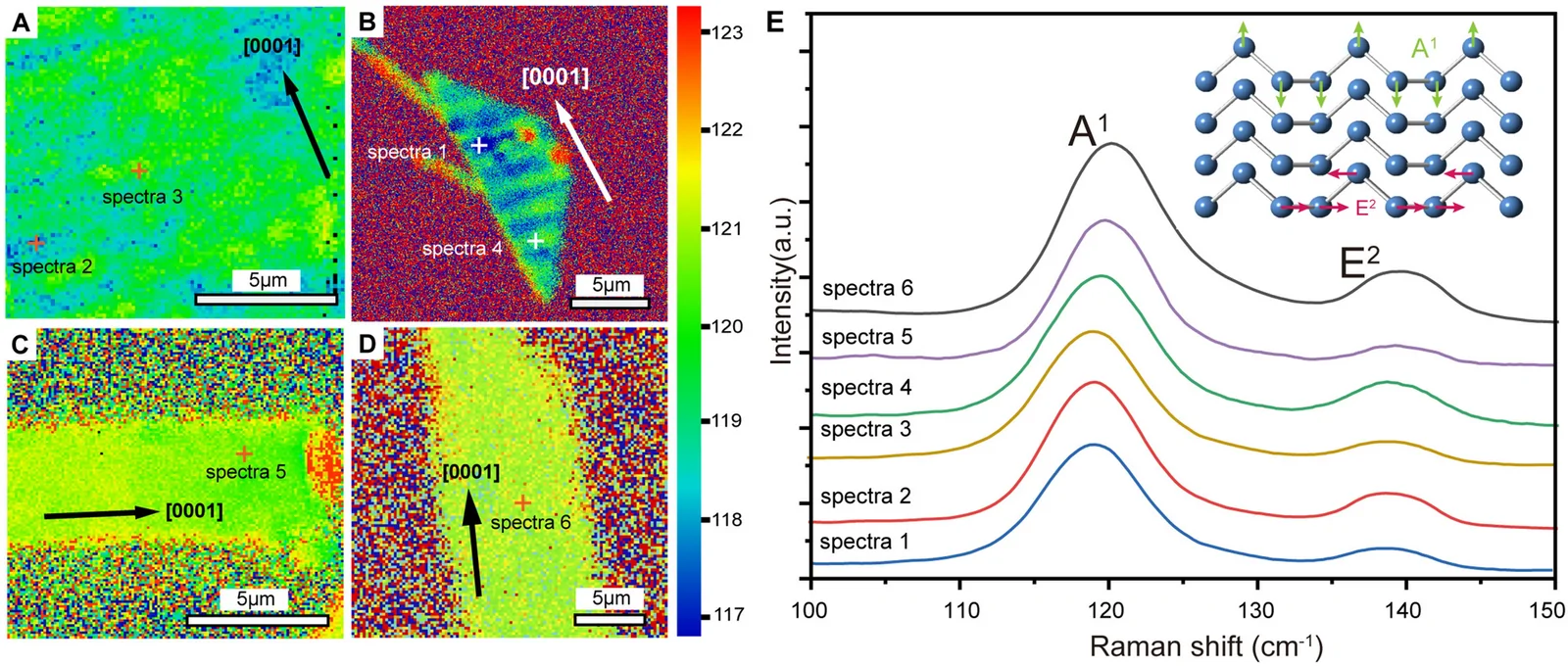
Raman mapping of the strained Te flake under A^1^ mode, **A** strain field induced by LSI on CD mold that is perpendicular to the chain direction, **B** strain field induced by LSI on grating mold oriented perpendicular to the chain direction, **C** strain field induced by LSI on CD mold that is parallel to the chain direction, **D** strain field induced by LSI on grating mold that is perpendicular to the chain direction, **E** Raman spectra of the strained 2D Te corresponding to the testing points in **A** to **D**

When the strain field is oriented perpendicular to the [0001] chains—such as in samples processed with a CD mold or when in contact with the sharp edges of a blaze grating mold—a pronounced red shift in the A^1^ mode is observed, shifting the peak to around 117 cm^−1^ (Fig. 4A, B, E). This red shift is indicative of a weakened interatomic interaction, suggesting that the lattice is in a state of tensile residual stress, as illustrated in Fig. 4A. Mechanistically, during LSI the aluminum layer transfer the shock wave into the Te flake and deform it into the nanoscale features of the mold. In regions where the trenches or grating edges press against the Te, the lattice is elongated perpendicular to the helical chains. The length of the Te–Te covalent bond was elongated and the interatomic interaction was weakened. The confined geometry and high pressure hinder the recovery of the elastic strain completely, resulting in a residual tensile stress that decreases the vibrational frequency of the breathing mode. Further Raman mapping of the strained Te flake reveals a consistent red shift in the A^1^ mode to 117 cm^−1^ in regions where the flake contacts the sharp edges of the blaze grating mold. This spectral shift, observed across different contact geometries (CD trenches and blaze grating edges), suggests a common mechanism of stress induction and lattice deformation. The red shift serves as a direct indicator of localized tensile stress, providing insights into the strain distribution within 2D Te. During ultrafast deformation, elastic energy is stored in lattice distortions and defects, generating tensile stress that weaken the breathing vibration in the $(10\overline{1 }0)$ plane, decreasing vibrational frequency.

On the other hand, when the strain field is aligned parallel to the [0001] helical chain direction, the Raman spectrum shows only subtle changes—a minor blue shift from the baseline peak (121 cm^−1^) to about 121.5 cm^−1^ in localized regions. This blue shift is consistent with previous reports ^9, 22^ and can be attributed to compressive strain, which enhanced the interatomic bonds along the chain. The weak van der Waals interactions between the chains facilitate chain slip during deformation without inducing significant tensile or compressive stresses along the chains. In this case, the predominant mechanism is chain gliding, which accommodates the external load while largely preserving the intrinsic vibrational characteristics of the helical structure. Following the rapid LSI process—an ultrafast compression pulse—residual tensile stress persists in the Te flake, retaining some elastic strain and causing the observed blue shift in the Raman spectrum. In Raman mapping of the strained Te for strain field aligning parallel to the Te chains (Fig. 4C-E), most of the flake exhibits a Raman shift at 120 cm^−1^, representing minimally strained regions. However, localized areas show a blue shift to ~ 121.5 cm^−1^, indicating compressive residual stress. The absence of significant Raman shifts in Fig. 4D suggests that certain parallel strain conditions do not disrupt interatomic interactions along the helical chains, preserving the breathing vibration mode in the $(10\overline{1 }0)$ plane. This deformation mechanism enables strain accommodation without altering fundamental vibrational characteristics of the chains. In contrast, perpendicular strain induces stronger tensile forces due to the covalent bonding along Te chains, preventing slip and leading to direct lattice elongation. The localized strain—concentrated at nanoscale mold boundaries—causes a heterogeneous distribution of elastic energy and defects, further amplifying compressive forces and producing a pronounced blue shift.

The contrasting spectral shifts—minor blue shifts under parallel strain and significant red shifts under perpendicular strain—highlight the crucial role of crystallographic orientation in 2D Te’s deformation response. Parallel strain primarily induces chain slip, preserving the vibrational signature with minimal perturbation. In contrast, perpendicular strain causes direct lattice elongation, significantly altering interatomic interactions and vibrational frequencies, leading to a pronounced red shift in the A^1^ mode. These findings underscore Te’s anisotropic mechanical behavior and provide a foundation for strain engineering strategies in 2D materials, where controlled modulation of lattice vibrations can enhance electronic and optoelectronic performance.

### Orientation-Dependent Deformation of 2D Te under Laser Shock: A Combined MD and Experimental Analysis

#### Simulation Methods

Molecular dynamics (MD) simulations of laser-induced shock in tellurium (Te) chains on a silicon dioxide (SiO₂) substrate require accurate force fields for Te–Te intrachain, Te–Te interchain, and Te–substrate interactions. As no existing force fields adequately describe these interactions, we developed in-house parameterized models following a hybrid approach similar to Branco and Cheng [26]. First, we employed self-consistent field (SCF) calculations using Quantum ESPRESSO to determine equilibrium and strained state energies of Te configurations. We modeled Te intrachain interactions in a hexagonal/trigonal unit cell and interchain interactions in a hexagonal arrangement, systematically varying atomic positions, and interatomic distances to obtain energy differences. Chain–substrate interactions were simulated in a triclinic unit cell containing a single Te chain atop SiO₂, varying the chain–substrate spacing.

The molecular dynamics simulations of tellurium (Te) chains shocked over silicon dioxide (*SiO*_2_) substrate depend on the existence of specific force fields for all the interatomic interactions within the system. Those interactions consist of Te–Te intrachain interactions, Te–Te interchain interactions, Te–O and Te–Si chain–substrate interactions. There are no force fields available to execute those interatomic interactions for the Tellurium chains. Therefore, we needed to develop force field model parameters in-house so we could perform the molecular dynamics laser-induced shock simulations.

**Fig. 5:**
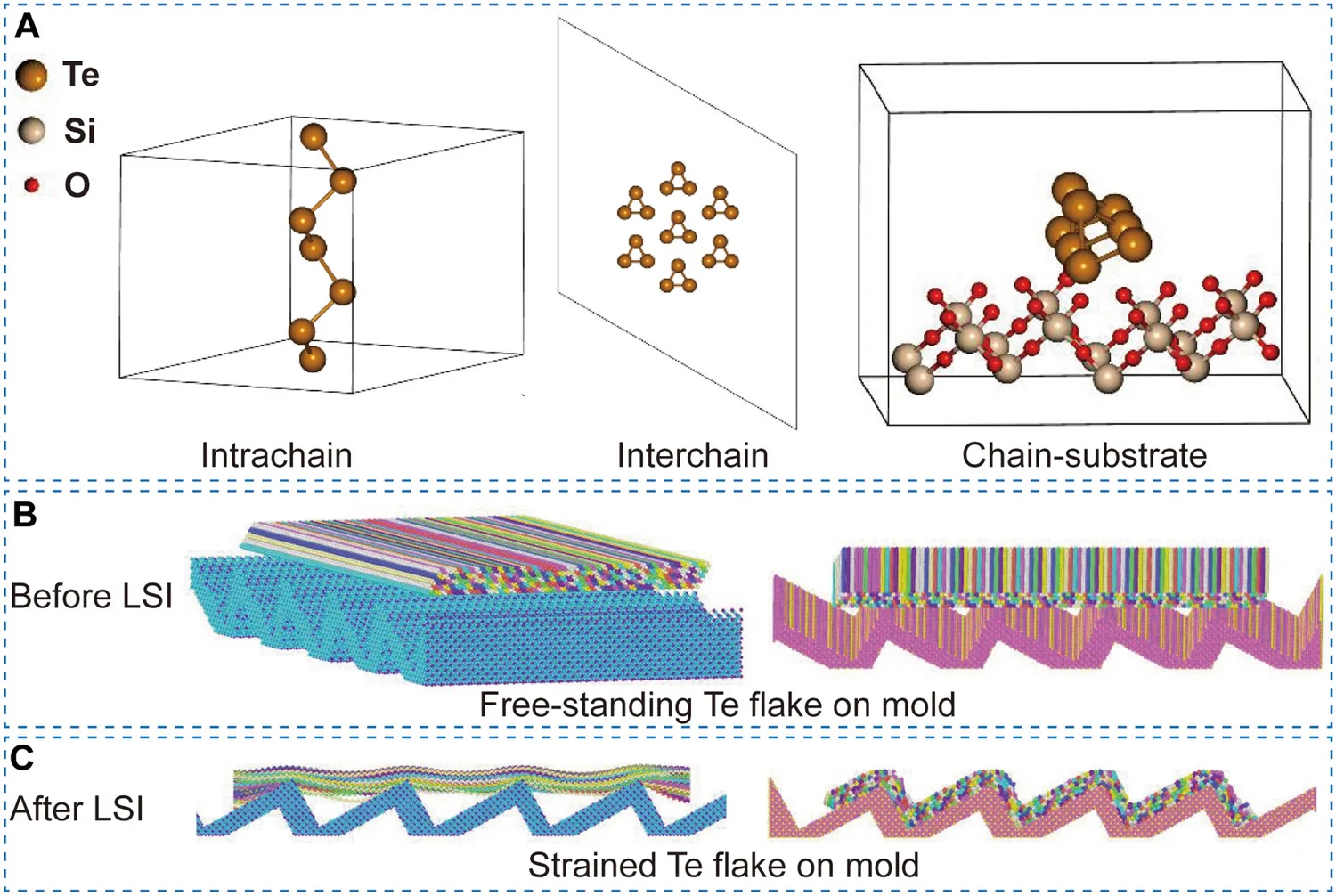
MD simulation of the asymmetrical strain engineering of tellurene on grating mold via LSI. **A** Types of simulation volumes for parametrization of interatomic interactions. The orange spheres represent the tellurium atoms, the red spheres represent the oxygen atoms, and the light tan spheres represent the silicon atoms. **B** Two employed orientations, transversal and parallel to gratings, for the Te chains shocked over the grating SiO_2_ substrate before the laser shocking. **C** Te chains conformed to the SiO_2_ grating substrate after the laser shocking

We developed the force field models using a procedure similar to the approach used by Branco and Cheng, 2021 [26] for developing force fields using a hybrid models approach. In this approach, we have started by executing self-consistency calculations, using the Quantum Expresso software [27] (Quantum Mechanics simulation tool), to determine the atomic system’s energy in the equilibrium state (Fig. 5A). Then, varying atomic positions, we have determined the system’s energy at strained conditions. After determining the system’s energy in Quantum Expresso, we used LAMMPS [28] (molecular dynamics simulation tool) and run MD simulations using the same configurations used in the Quantum Expresso’s simulations. For each system configuration, we run different combinations of parameters for Lennard–Jones, harmonic angle, and harmonic dihedral models’ interactions. Finally, we have compared the differences between the Quantum Expresso’s systems’ energy results with the differences obtained by the LAMMPS system’s energies results. The parameters set that resulted in the smallest differences errors (Fig. S11), was the chosen parameters set for the interatomic interactions models (Table S1).

We have initiated the parametrization process by determining systems’ energies of some specific atomic configurations containing the types of interatomic interactions to be parametrized. For that, we used the Burai software (quantum mechanics software that uses Quantum Expresso systems’ energy calculation). We have performed self-consistent field (SCF) calculations to find the energy of the simulated states for all tested atomic configurations. We started by modeling the Te chains intrachain interactions. We first imported the Te chains structure from [4] and changed the unit cell dimensions such that the unit cell contained 6 atoms in it (the 7th atom on top of the simulation cell in Fig. 5A only shows the periodicity of the unit cell, but is not contained in the cell), so all types of intrachain interactions could be later simulated in the molecular dynamics environment and compared to the SCF results. A hexagonal and trigonal unit cell with the dimensions A = 12.0248 Å and C = 11.4000 Å was employed and a 5 × 5 × 7 K-points simulation space was set. The A value was set to a large value so periodic boundary condition effects in the X and Y directions did not affect the chain potential energy determination. A Perdew–Burke–Ernzerhof (PBE) exchange functional, a wave function cutoff of 25.000 Ry, and a charge cutoff of 225.000 Ry were applied to the simulation. The unit cell C dimension along with the Te atoms positions were altered and the total system’s potential energy was determined for 6 different configurations. The energy differences between each system energy and the state of minimum energy through the different C lengths were determined (Fig. S11A). A similar procedure was executed for the Te interchain potential energy determination. The same type of unit cell was employed but with dimensions A = 25.5371 Å, and C = 10.9800 Å. However, instead of varying the unit cell dimensions along with atomic positions, only the atomic positions were varied this time to form the different systems. The Te chains in this system formed a regular hexagon with chains in the vertices and in the center of the hexagon (Fig. 5A). The distances between the Te chains were then varied between each simulated system. However, the chains disposition was still in the regular hexagon shape with a chain in the middle of this polygon. For the interchain interactions, 7 atomic states/systems were simulated, and the energy stets differences were determined (Fig. S11B). The system to determine the chain–substrate interactions was built in a triclinic unit cell (Fig. 5A) with dimensions A = C = 16.0000 Å and B = 12.0000 Å, containing a single Te chain and the top layer atoms of a *SiO*_2_ crystal. The same exchange functional, wave function cutoff, and charge cutoff from the interchain and intrachain interactions were employed in this chain–substrate system. 6 systems/states were simulated and had their potential energy determined. The only factor that varied between these systems was the distance between the Te chain and the silicon dioxide’s surface. The system’s energies differences were also determined for different chain–substrate spacing (Fig. S11C).

To simulate the Te chains intrachain interactions, we chose two-, three-, and four-body interactions since higher-order interactions do not have significant contribution to the system’s total energy. The two-body interactions were modeled using the Lennard–Jones model (Eq. 1). The three-body interactions were modeled using the angular harmonic model (Eq. 2), and the four-body interactions were modeled using the dihedral harmonic model (Eq. 3).

1 $$\begin{array}{c}E=\sum\limits_{i}\sum\limits_{j>i}4\epsilon \left[{\left(\frac{\sigma }{{r}_{ij}}\right)}^{12}-{\left(\frac{\sigma }{{r}_{ij}}\right)}^{6}\right],{r}_{ij}<{r}_{c}\end{array}$$

where *r*_*ij*_ is the distance between atoms i and j, and *rc* is the cutoff radius (*rc* = 9 Å in this study).

2 $$\begin{array}{c}E={C}_{\alpha }{\left(\theta -{\theta }_{0}\right)}^{2}\end{array}$$

where θ is the angle between 3 Tellurium atoms.

3 $$\begin{array}{c}E={C}_{D1}\left[1+\mathit{cos}\left({c}_{D2}\phi \right)\right]\end{array}$$

where ϕ is the dihedral angle formed between a triplet plane of consecutive Tellurium atoms in a chain and the bond to an immediate Tellurium neighboring atom to this triplet.

The interchain and chain–substrate interactions were modeled using only two-body interatomic interactions, employing the Lennard–Jones model. Therefore, the models used to simulate the interatomic interactions presented 12 different parameters (Table 1) to be determined by the parametrization process.

Just like what was done in the first principles calculations, we have performed separate parametrization steps for the components of the atomic system. For the intrachain interactions, which contained 6 parameters, we created 10^6^ parameters sets to be tested in MD for each chain state simulated in force field model’s parametrization. We performed MD simulations using the same simulation box geometry as the ones applied to the Burai simulations. Then we have also replicated the atomic positions in the simulation volume with periodic boundary conditions for all the simulated chain states. An NVT ensemble with temperature of 0.1 K was set and a single times tep was run for each atomic configuration since the potential energy of the system was the goal in this simulation. The parameters set that resulted in the closest approximation to the SCF results (highest coefficient of determination *r*^2^) was the chosen parameter set (Table S1). A similar procedure was done to determine the 2 parameters for the interchain interactions, and for the 4 remaining parameters of the chain–substrate interactions.

#### *Structural Evolution of 2D Tellurium *via* MD Simulations*

After determining the interatomic interaction parameters, molecular dynamics (MD) simulations were conducted using LAMMPS to model the laser shock process on 2D tellurium (Te) flakes. Simulations were performed in an NVT ensemble at room temperature with periodic boundary conditions, using a time step of 1 fs. A silicon dioxide substrate was constructed to mirror the aspect ratio of the experimental molds, and a Te chains flake (dimensions: 450 × 220 × 17.74 Å^3^) was placed atop the substrate. A shock pressure—defined by the load profile in Fig. S12—was applied for two distinct flake orientations relative to the mold, as depicted in Fig. 4.

Upon completion of the shock, the Te flakes adopted markedly different configurations (Fig. 5B, C), revealing fundamental insights into the deformation mechanisms driven by the orientation of the Te chains relative to the applied stress. In the case when Te chains are oriented transversely to the gratings, the applied stress induces a pronounced twisting of the chains. This twisting, which deviates significantly from the relaxed state, is confirmed by high-stress orientation reversals in HRSTEM images (Fig. 3), underscoring a three-dimensional deformation response where the chains resist direct compression by reorienting their local structure.

Conversely, when the Te chains are aligned parallel to the gratings, the deformation mechanism is dominated by extensive chain sliding in high-stress regions. This sliding leads to significant rearrangement of interchain spacing relative to the relaxed configuration, indicative of the inherent anisotropy of 2D Te. The lower resistance to sliding along the chain direction facilitates a reorganization that disrupts the ordered alignment seen in the unstrained state. Such configurational changes manifest in TEM images as blurring, reflecting a heterogeneous mixture of chain orientations and a loss of the characteristic ordering observed in the pristine material. The absence of a uniform chain orientation in these regions results in a more disordered local structure, which manifests as reduced image clarity in HRSTEM and TEM analysis.

A better understanding, in quantitative terms, of the effect of different loading scenarios on the deformation mechanisms can be obtained via MD simulations. In the simulation, 64 Te chains 27.8 Å long were assembled into the simulation volume. The same Te chains MD simulations setup (used for the qualitative defects formation analysis of the shocked Te chains onto the trenched substrate) but without a trenched substrate was built and three different loading cases were simulated.

The first loading case was a compressive-to-tensile loading along the Te chains longitudinal orientation. This loading case was used as a baseline to verify the tensile and compressive stress levels at which the chains failed. This loading case consisted of applying forces on each of the top atoms, while the bottom atoms were fixed in place, (in the z-axis only) along the longitudinal direction of all the Te chains in the simulation volume, as shown in Fig. S14 (A), 25,000 time steps were executed before applying the loads. Then the longitudinal loads were applied on the top atoms and 50,000 time steps were executed at a temperature of 300 K to equilibrate the loaded condition at each loading level in the compressive-to-tensile simulations. The stress–strain response along the longitudinal loading direction is shown in Fig. S14B. Chains breakage was observed for loading levels outside of the simulated limits (Fig. S14C). From the simulations results, as shown in Fig. S14 it was possible to determine that the modulus of elasticity of the Te chains along their longitudinal orientation is E=92.26GPa. Therefore, chains breakage happens at stress levels of magnitude higher than 2.4GPa. In addition, no chains gliding or twisting were observed through the longitudinal loading simulations.

The second loading case was a shear loading in the $(01\overline{1 }0)$ plane. This loading case was used to obtain the shear stress level in the $\left(01\overline{1 }0\right)$ plane for which chains gliding and twisting happen. Studying this loading case is important to understand when and how the Te chains twisting and gliding happen and if this is a preferred orientation for these defects’ formation. This loading case where the simulation steps were similar to the ones from the first loading case simulation, consisted of applying forces in the x-axis only on the top half atoms of the simulation volume (transversal loads on the 32 top chains of the simulation volume), as shown in Fig. S15A.

The shear stress at which chains gliding on the $(01\overline{1 }0)$ plane started occurring was 460MPa. Just prior to chains gliding and combined with chains gliding Fig. S15C), chains twisting was also observed as shown in Fig. S15B.

The third loading case was a compressive-to-tensile loading in the $(01\overline{1 }0)$ plane. This loading case was used to obtain the normal stress level in the $(01\overline{1 }0)$ plane for which chains gliding and twisting happen and if any chain breakage can occur before chains twisting and gliding. Studying this loading case is important to understand when and how the Te chains twisting and gliding happen and in which plane they happen when this loading case is applied this is a preferred orientation for those defects’ formation. This case where the simulation steps were similar to the ones from the first loading case simulation, consisted of applying forces in the y-axis only on the top atoms of the simulation volume (transversal loads on the top chains of the simulation volume), as shown in Fig. S16.

The complex loading state generated through shocking Te chains onto a trenched substrate generates compression and tensile loads on all axes as well as shear loads. This complex loading state induces the deformation mechanisms and causes chains gliding as well as twisting depending on the shock pressure and the stress state it develops. Although MD simulations may overestimate loading levels for defects formation due to size effects, strain rate, and lack of initial defects, the quantitative analysis through MD simulations is important to show which deformation and defects formation mechanism happens preferably according to the loading case. These simulation results, when correlated with experimental observations, provide deep mechanistic insights into the anisotropic response of 2D Te under ultrafast laser shock conditions. The twisting observed in transversely oriented flakes suggests that the applied compressive load is partially mitigated by a rotation of the Te chains, a mechanism that may help dissipate localized stress. In contrast, the chain sliding in parallel orientations highlights a distinct deformation mode where the intrinsic weak interchain interactions promote lateral displacement rather than rotational accommodation. These variations induce modifications in local atomic configurations and can be detected via altered imaging contrast in high-resolution scanning/transmission electron microscopy (HRSTEM), necessitating the advanced characterization techniques to resolve these subtle, crystallographic orientation-dependent structural distortions with precision. Understanding these mechanisms is crucial for predicting and controlling the behavior of 2D Te under various stress conditions, which is essential for its potential applications in flexible electronic and strain-engineered devices. By linking simulation outcomes with experimental imaging, this study provides a comprehensive picture of how external stress, when applied under controlled conditions, can drive complex, orientation-dependent lattice distortions in 2D materials.

## Conclusions

In conclusion, this study explores the fundamental mechanisms underlying laser shock imprinting (LSI) of two-dimensional (2D) chiral chain tellurium (Te), establishing it as a transformative, high-resolution, and ultrafast technique for achieving precise control over nanoscale straining in chiral chain 2D materials. By harnessing high-pressure shock waves generated through confined laser–material interactions, LSI enables the manipulation of Te's helical chain structures under ultrafast strain rates with unprecedented precision. The key findings reveal two distinct deformation regimes based on the orientation of the applied strain field relative to the helical chains. When the strain field aligns parallel to the helical chains, deformation primarily occurs through chain slippage and rotation, introducing lattice defects without significantly altering the material's Raman response, as the vibrational characteristics of the chains remain intact. This underscores the robustness of Te's covalent intrachain bonding under strain. In contrast, perpendicular strain fields induce a complex interplay of chain rotation, slippage, stretching, and bending. Symmetrical strain fields result in uniform wavy dislocations along the helical chain direction, while asymmetrical strain fields generate high-density dislocation tangles in localized regions. Remarkably, areas subjected to asymmetrical strain can retain single-crystal structures, demonstrating Te's ability to accommodate localized deformation without compromising structural integrity. These findings provide a fundamental contribution to the understanding of deformation mechanisms in chiral chain 2D materials under extreme conditions. The ability to precisely control strain-induced structural changes in Te opens new avenues for tailoring its electronic, optical, and mechanical properties, with significant implications for applications in flexible electronics, optoelectronics, and strain-sensitive sensors.

## Supplementary Information

Below is the link to the electronic supplementary material.

Supplementary file1 (DOCX 9481 KB)
