# Supplementary material for: Ultrafast Laser Shock Straining in Chiral Chain 2D Materials: Mold Topology-Controlled Anisotropic Deformation
Source: Nanomicro Lett. 2025 Nov 19;18:83. doi: 10.1007/s40820-025-01925-8 (PMC12627285; doi:10.1007/s40820-025-01925-8)
Supplement: Supplementary file 1 — Supplementary file1 (DOCX 9481 KB) [file 40820_2025_1925_MOESM1_ESM.docx]

Supporting Information for

**Ultrafast Laser Shock Straining in Chiral Chain 2D Materials: Mold-Topology-Controlled Anisotropic Deformation**

Xingtao Liu^1,4 †^, Danilo de Camargo Branco^2†^, Licong An^3,4^, Mingyi Wang^1,4^, Haoqing Jiang^1^, Ruoxing Wang^1,4^, Wenzhuo Wu^1,4^* and Gary J. Cheng^1,3,4^*

^1^ School of Industrial Engineering, Purdue University, West Lafayette, IN 47906

^2^ School of Aeronautics and Astronautics, Purdue University, West Lafayette, IN 47906

^3^ School of Materials Engineering, Purdue University, West Lafayette, IN 47906

^4^ Birck Nanotechnology Center, Purdue University, West Lafayette, IN 47906

^†^ Xingtao Liu and Danilo de Camargo Branco contributed equally to this work.

*Corresponding authors. E-mail: [wu966@purdue.edu](mailto:wu966@purdue.edu) (Wenzhuo Wu); [gjcheng@purdue.edu](mailto:gjcheng@purdue.edu) (Gary J. Cheng)

**Supplementary Figures and Tables**


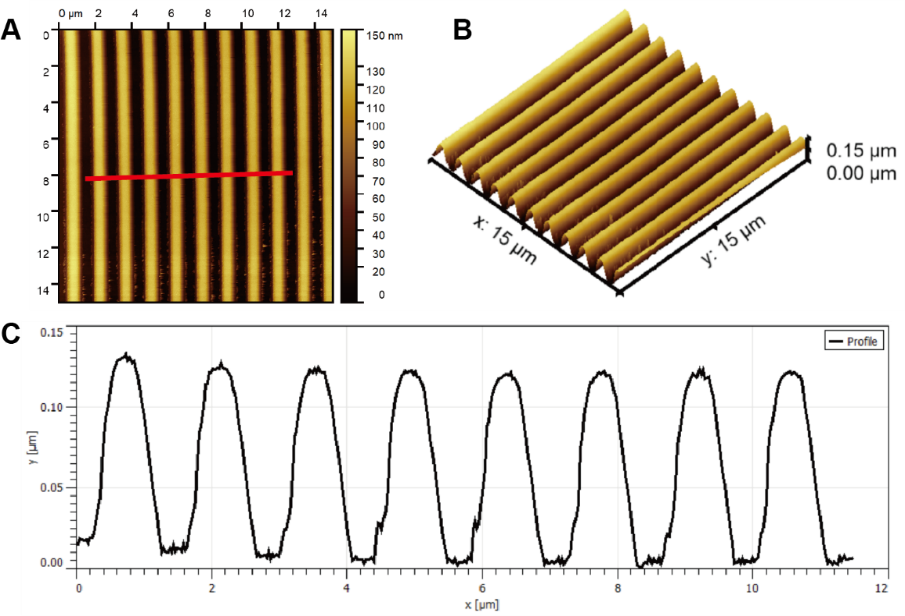


**Fig. S1** (**A**) AFM analysis of the CD molds; (**B**) 3D topography of the CD trenches in (A); (**C**) the height measurement of the red line in (A)


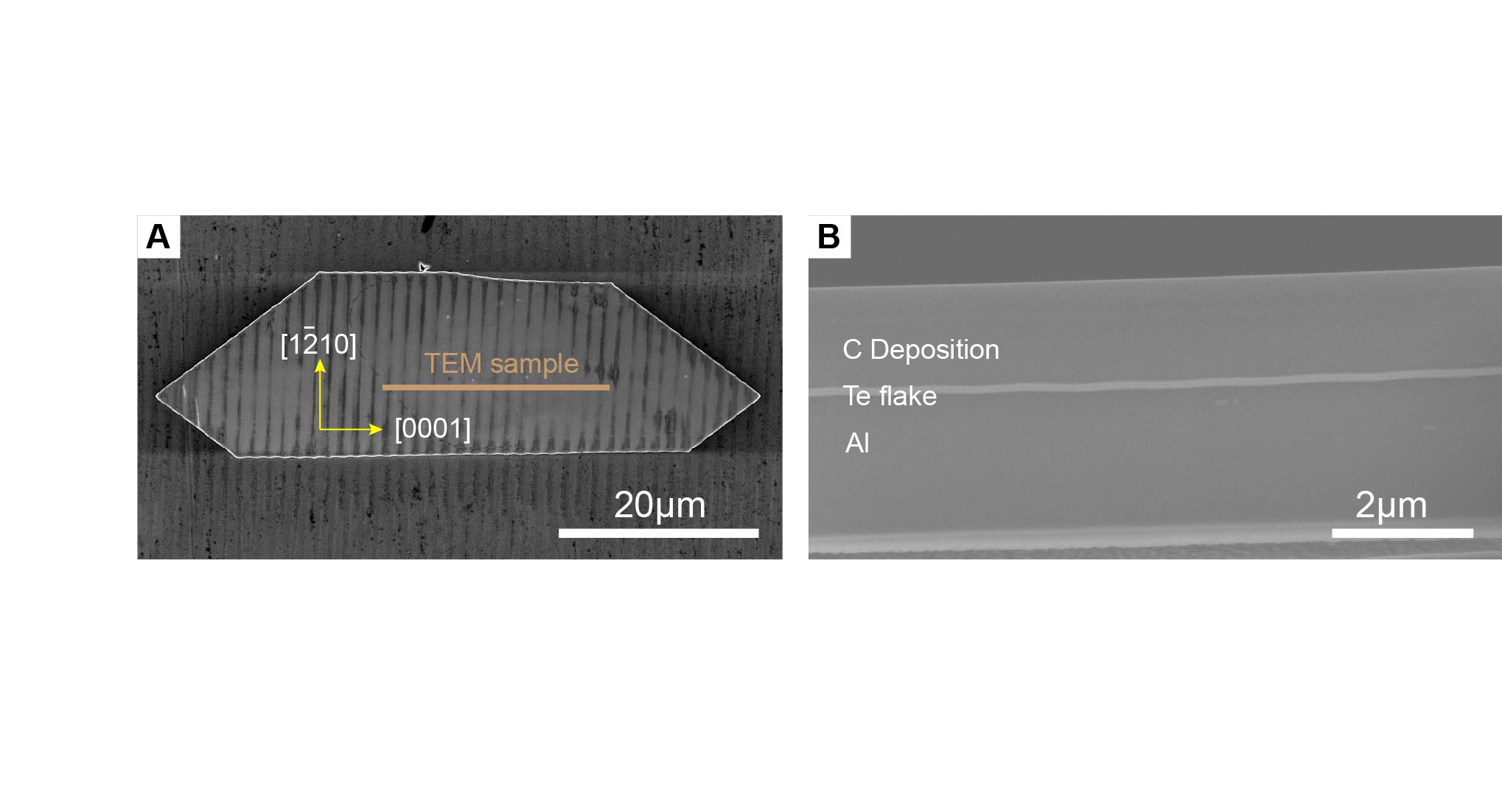


**Fig. S2** (**A**) SEM image showing the pattern on 2D Te and demonstrating the area of TEM sample. (**B**) FIB cross section imaging showing the morphology of the strained 2D Te on CD trenches that are perpendicular to the Te chains


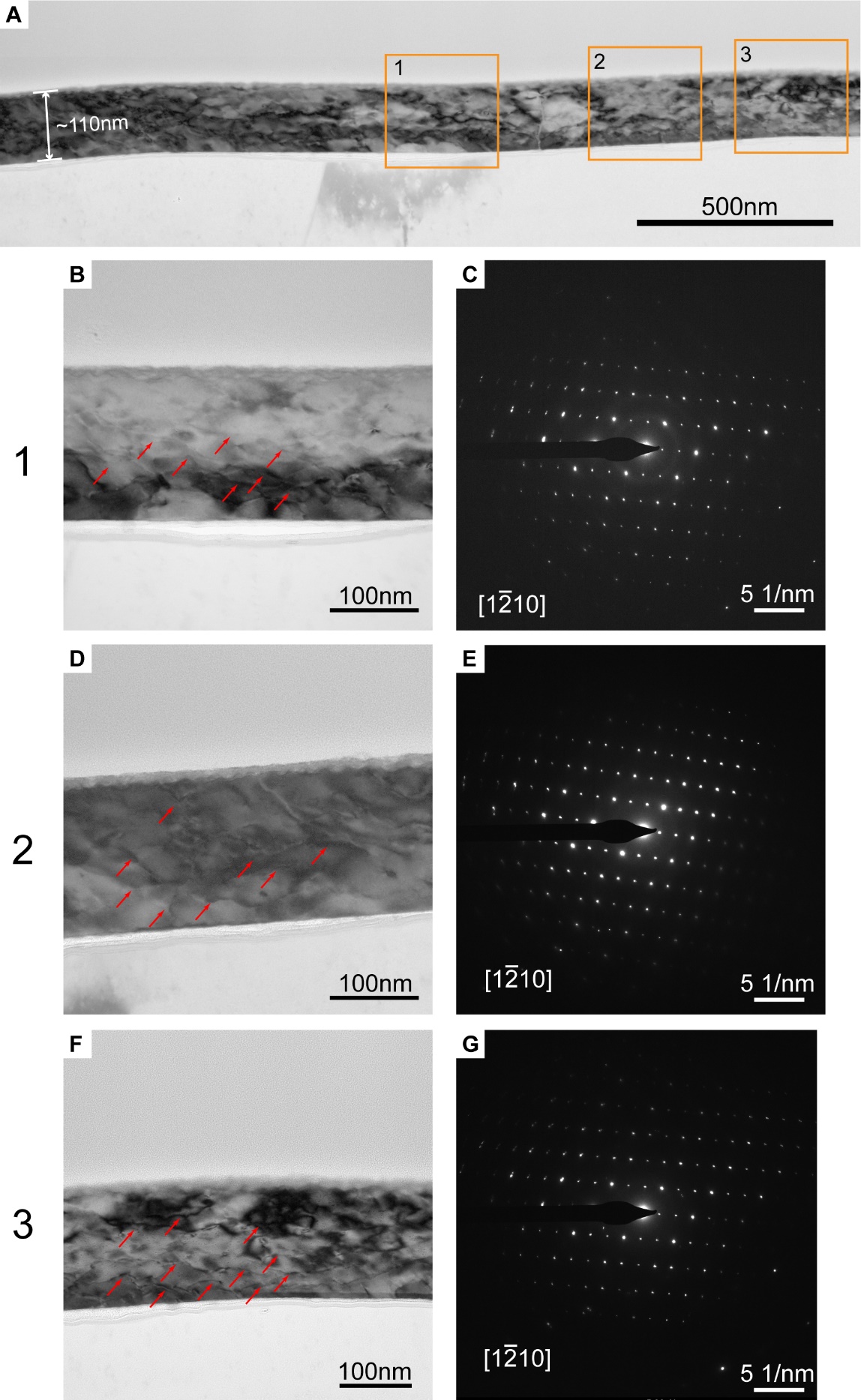


**Fig. S3** TEM analysis of the strained Te flake from CD mold, the strain field is perpendicular to [0001] direction. (**A**) Bright field TEM image of the strained Te (**B, D** and **F**) magnified images of the areas in (A). (**C, E** and **G**) corresponding SAD patterns of (B, D and F)


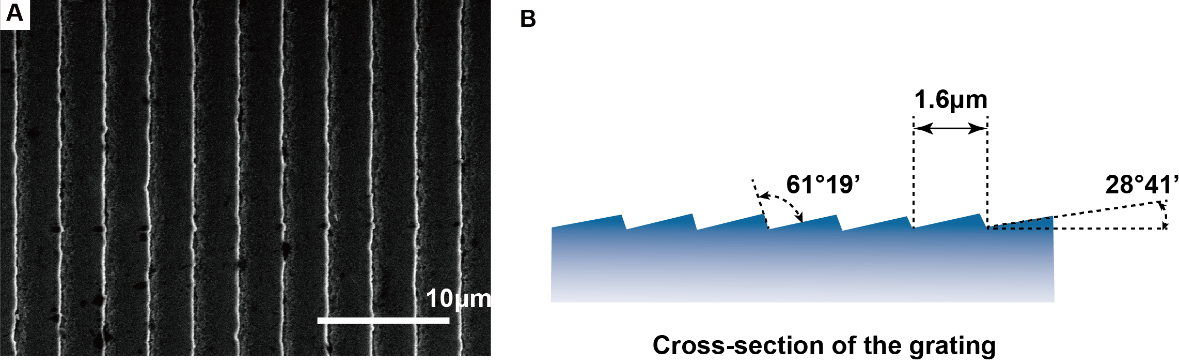


**Fig. S4** (**A**) SEM image of the grating; (**B**) the schematic showing the blaze wavelength and blaze angle of the grating


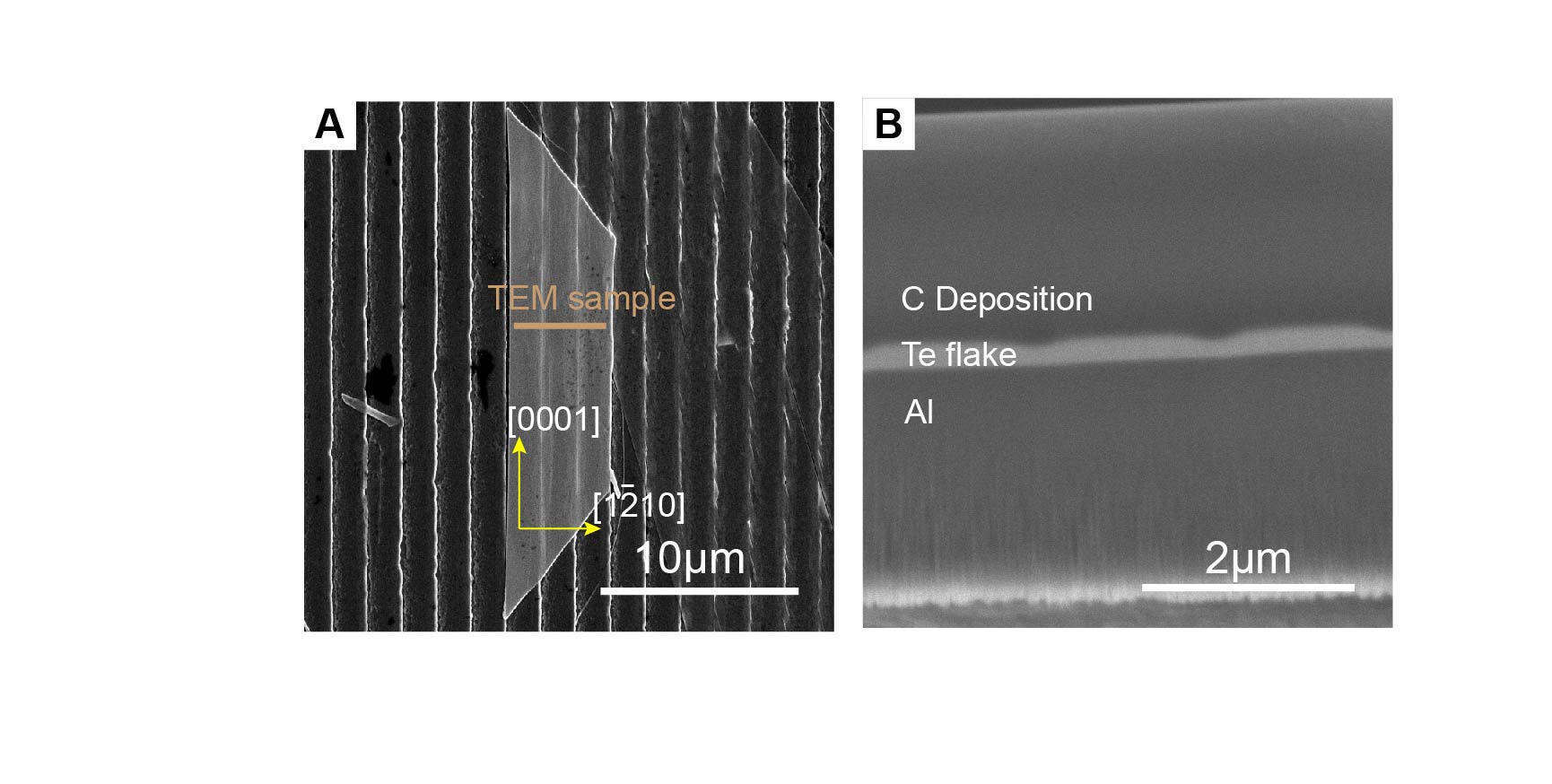


**Fig. S5** (**A**) SEM image showing the pattern on 2D Te and demonstrating the area of TEM sample. (**B**) FIB cross section imaging showing the morphology of the strained 2D Te on grating mold on which the sharp edges are parallel to Te chains


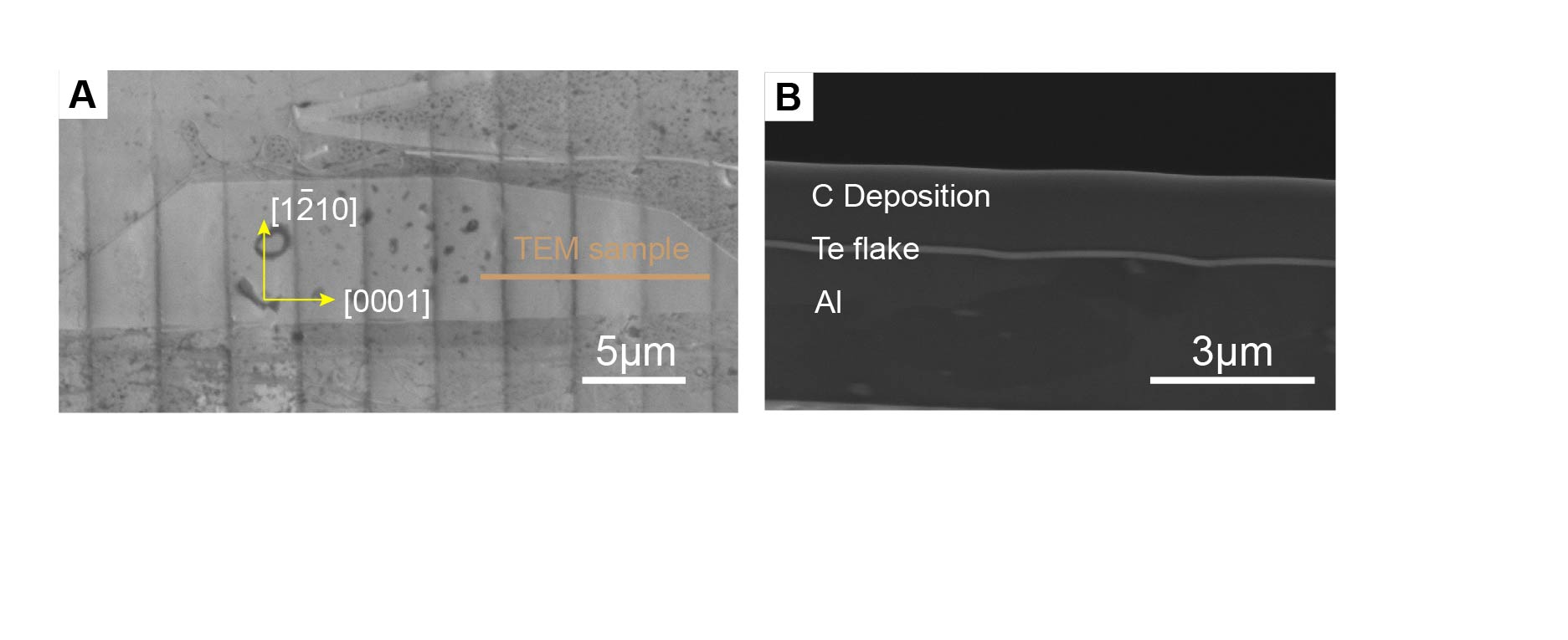


**Fig. S6** (**A**) SEM image showing the pattern on 2D Te and demonstrating the area of TEM sample. (**B**) FIB cross section imaging showing the morphology of the strained 2D Te on grating mold on which the sharp edges are perpendicular to Te chains


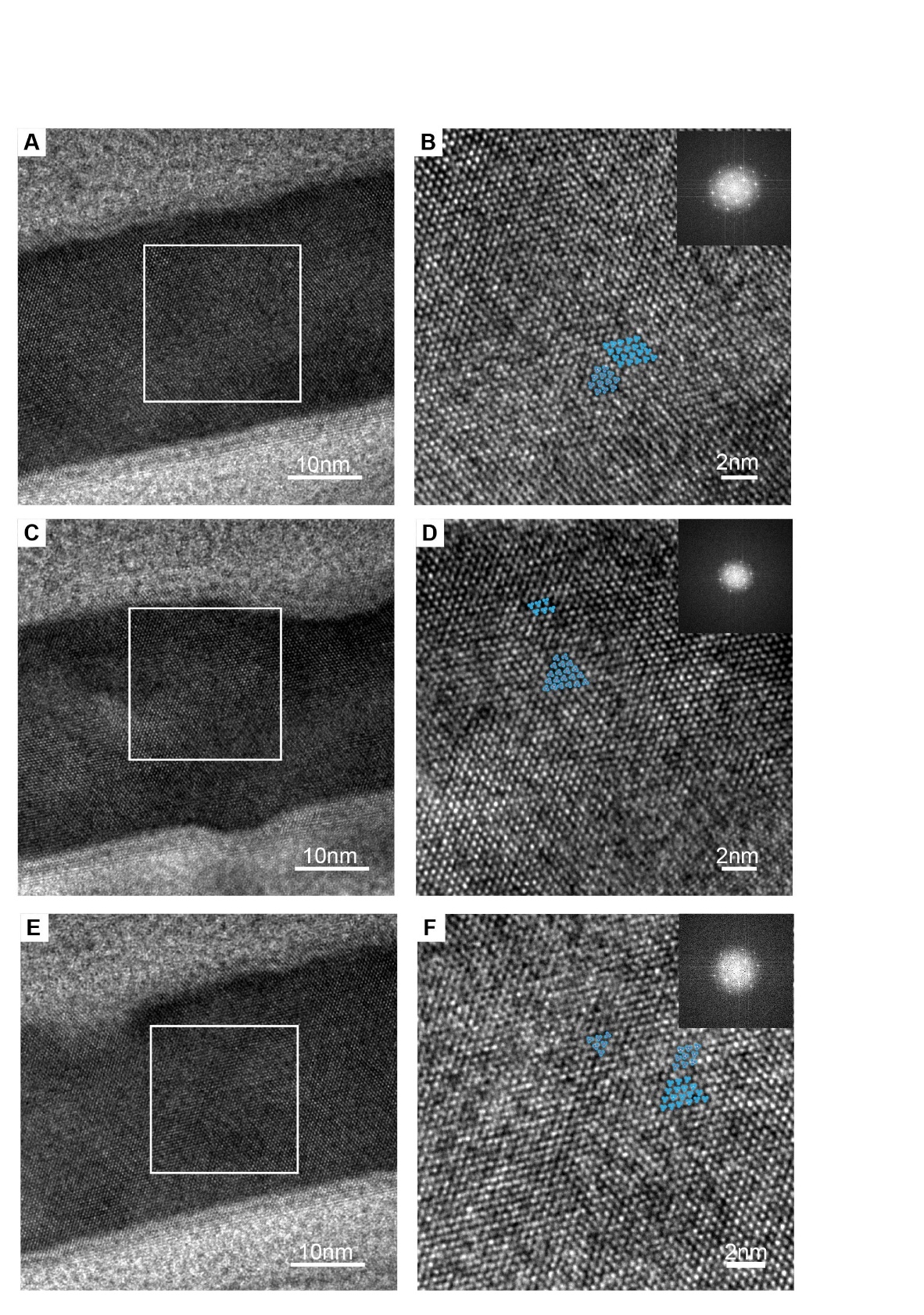


**Fig. S7** HRTEM images of the strained Te (**A-B**) HRTEM images and the magnified images of the area 1 in 1H (**C-D**) HRTEM images and the magnified images of the area 2 in **Fig.** 1H (**E-F**) HRTEM images and the magnified images of the area 3 in **Fig.** 1H. The image in top right corner of B, D and F are the corresponding FFT images


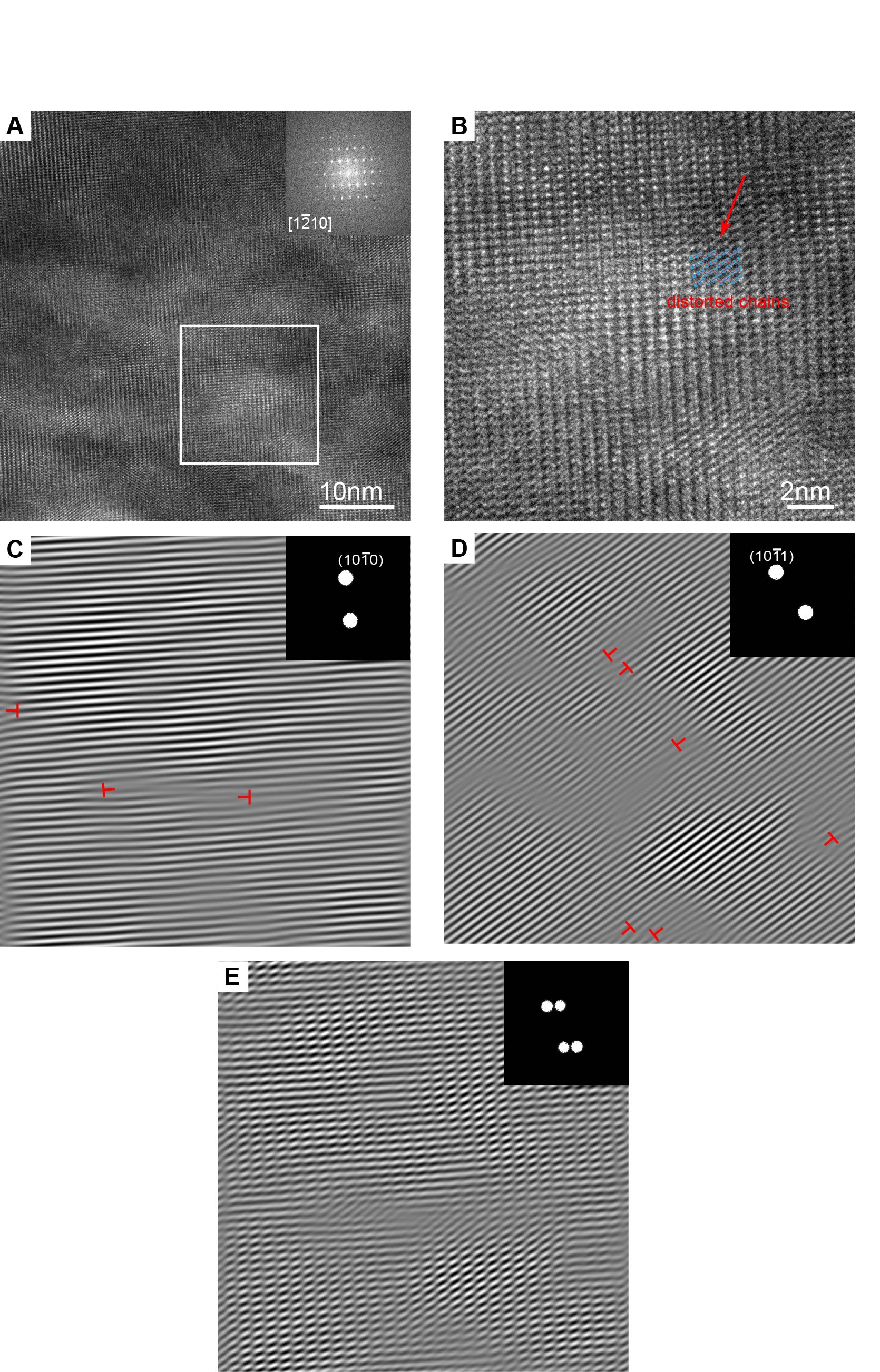


**Fig. S8** HRTEM analysis of the symmetrical strained Te flake on CD mold when the trenches are perpendicular to Te chains (**A**) HRTEM image, the FFT in the image is the FFT of the white square area. (**B**) The magnified image of the white square area in A; (**C-E**) The IFFT images of the masked areas in the FFT patterns


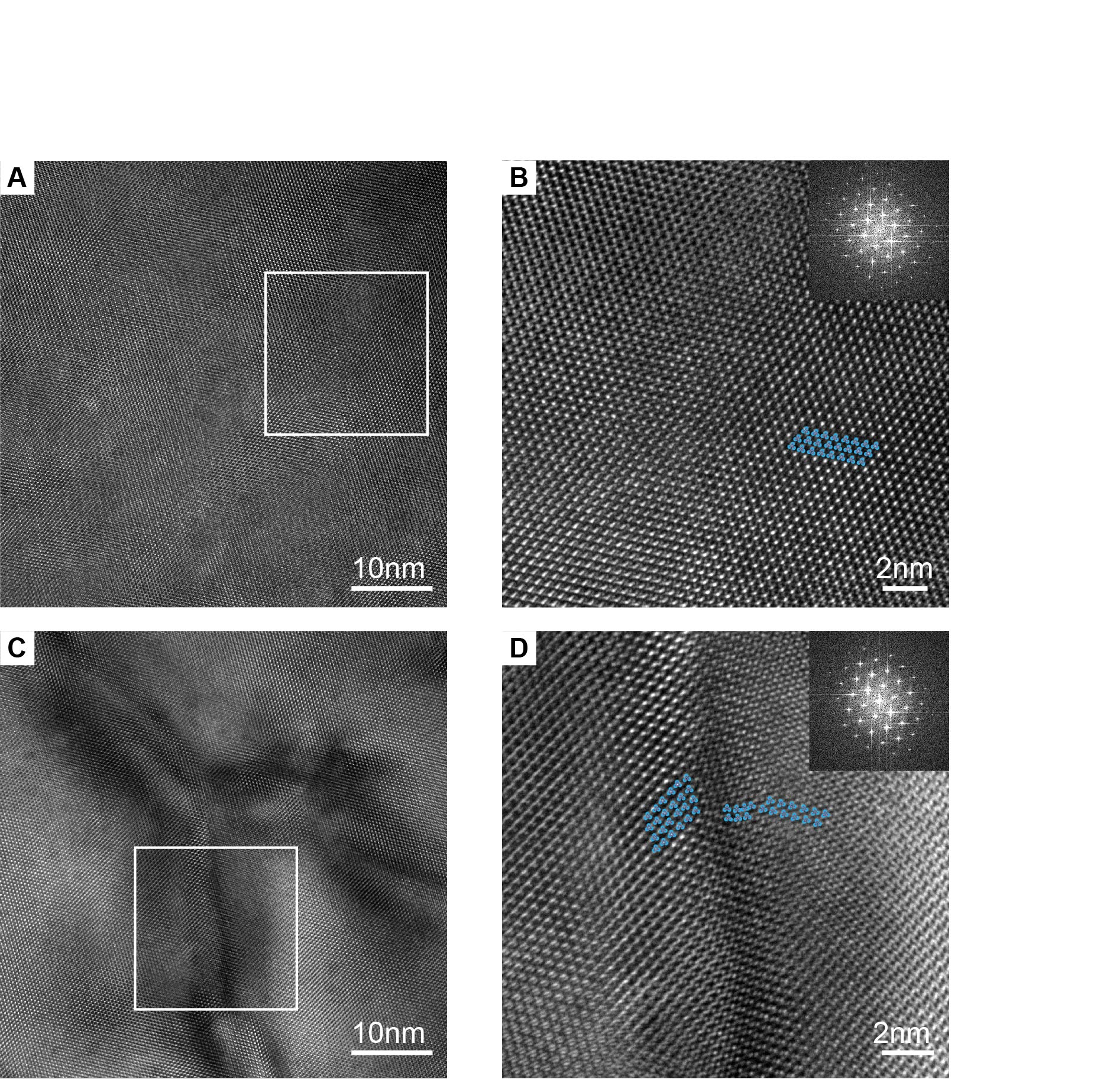


**Fig. S9** HRTEM analysis of the is asymmetrically strained Te flake on blazed grating mold, the grating edges are parallel to the [0001] direction. (**A**) HRTEM image of the flat area of Te flake (**B**) The magnified image of the white square area in (A) and corresponding FFT pattern (**C**) HRTEM image of the severely deformed area on Te flake (**D**) The magnified image of the white square area in (C) and corresponding FFT pattern


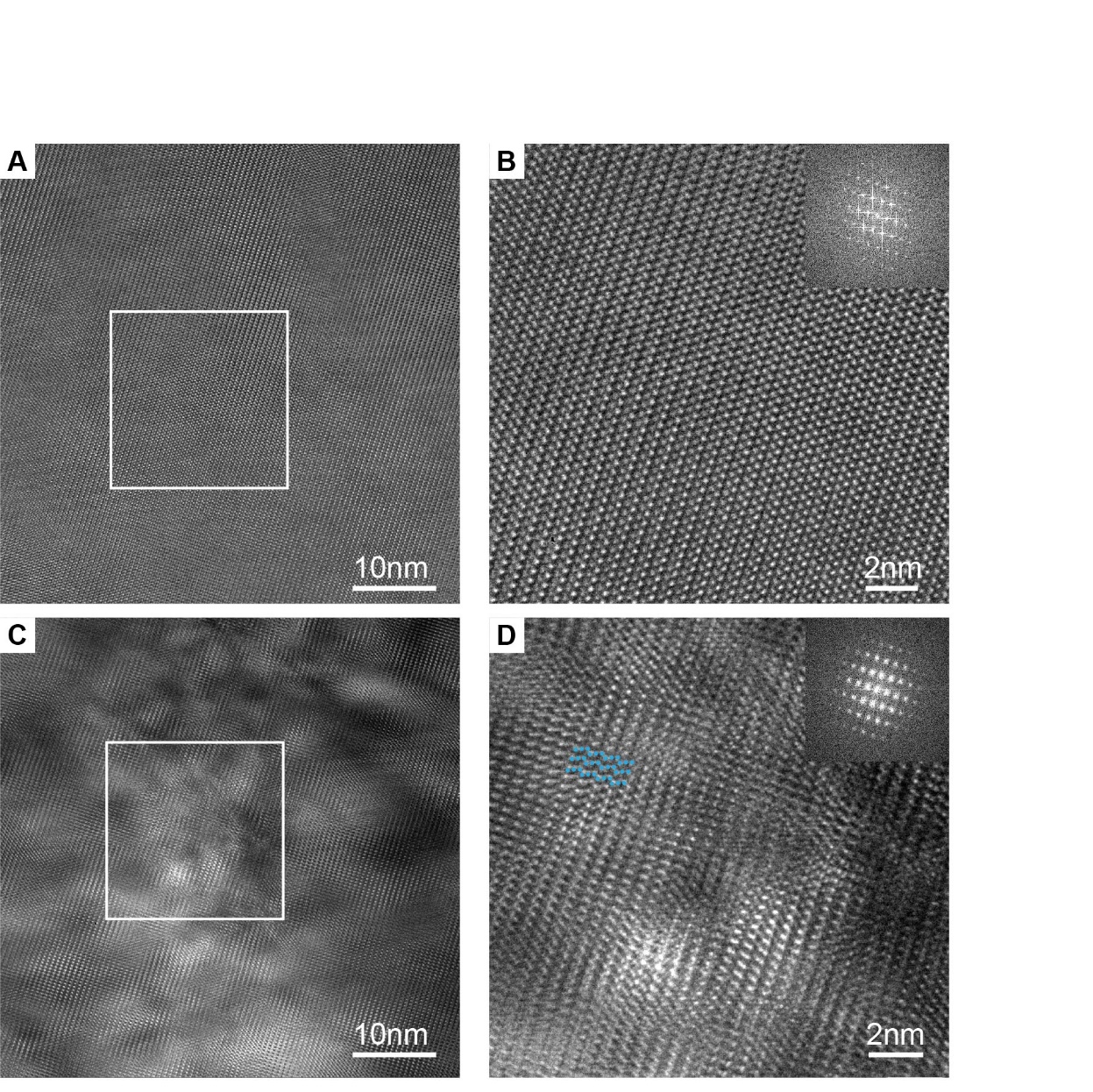


**Fig. S10** HRTEM analysis of the is asymmetrically strained Te flake on blazed grating mold, the grating edges are perpendicular to the [0001] direction. (**A**) HRTEM image of the flat area of Te flake (**B**) The magnified image of the white square area in (A) and corresponding FFT pattern (**C**) HRTEM image of the severely deformed area on Te flake (**D**) The magnified image of the white square area in (C) and corresponding FFT pattern


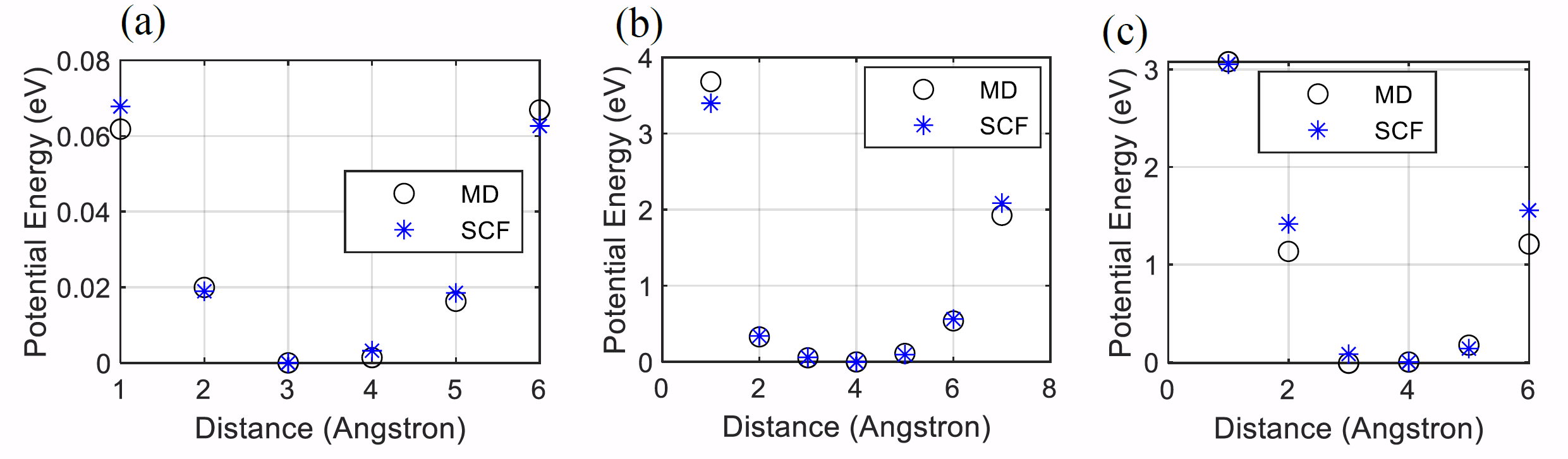


**Fig. S11** (**a**) Intrachain interactions. (**b**) Interchain interactions. (**c**) Te chain and 𝑆𝑖𝑂2 substrate interactions


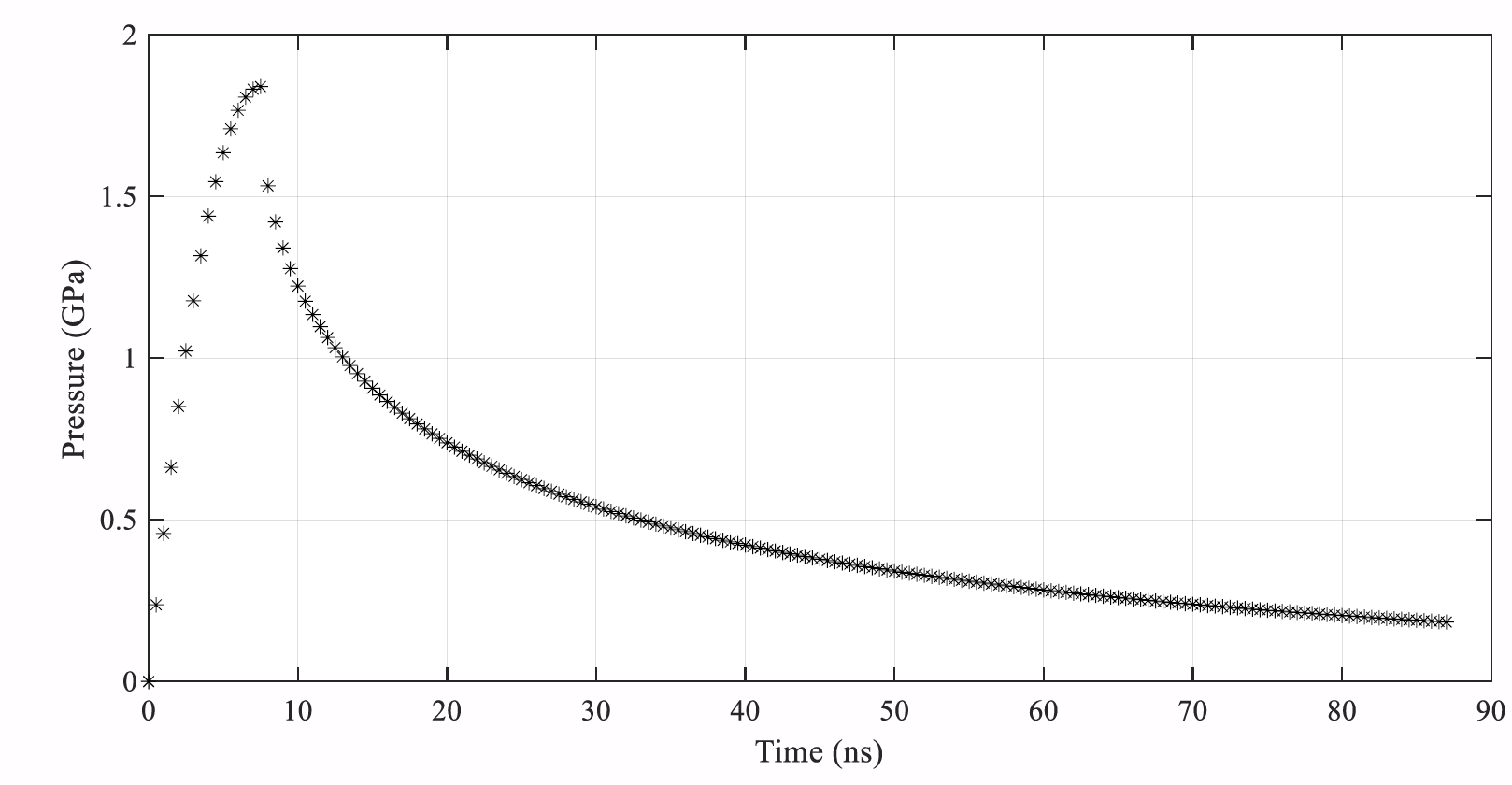


**Fig. S12** Shock pressure time history


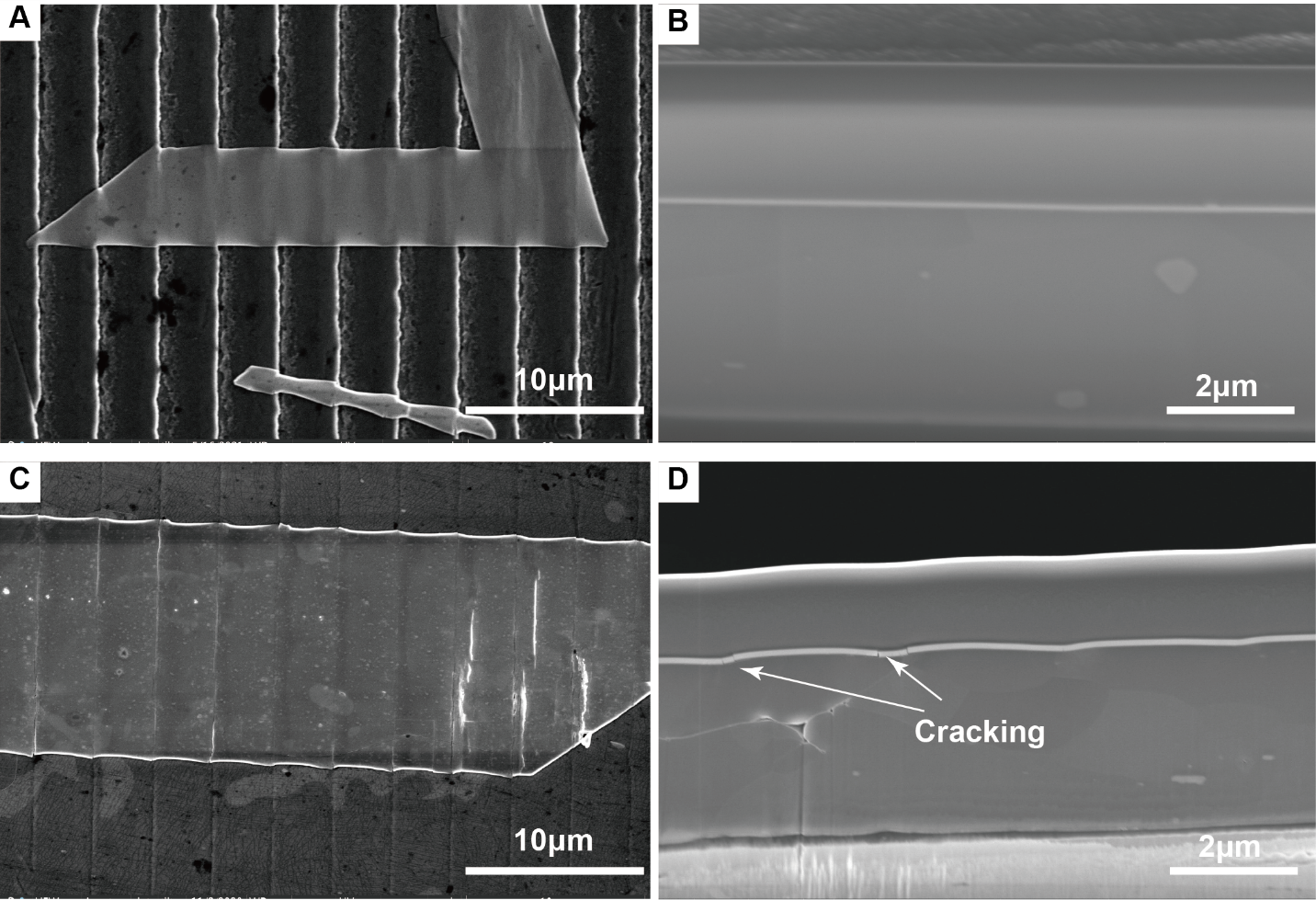


**Fig. S13** SEM and FIB cross section images of the Te flakes on blazed grating with different laser power density. (**A-B**) 0.1GW/cm^2^ (**C-D**) 1.12GW/cm^2^


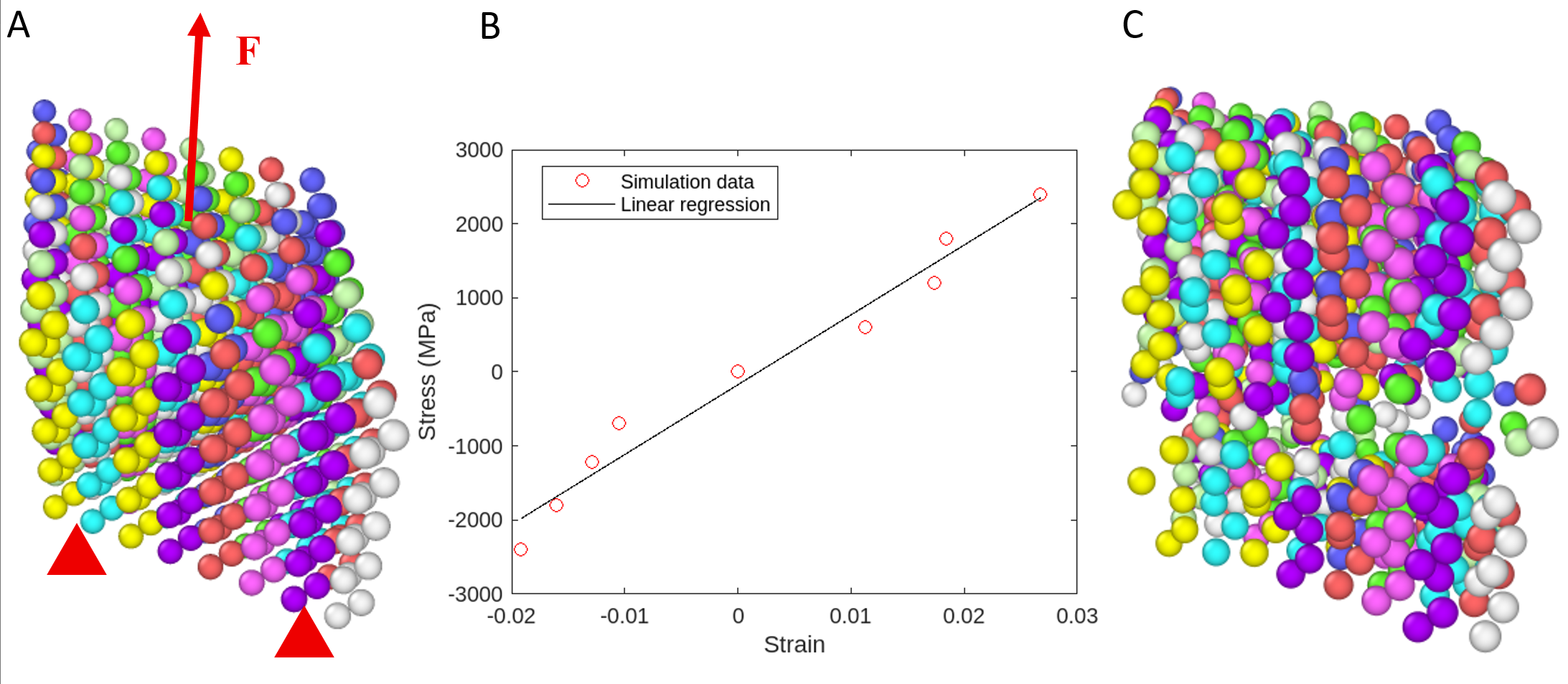


**Fig. S14** (**A**) Longitudinal loading setup for the first loading case where the top atoms are loaded on their longitudinal direction, and the bottom atoms are fixed in place; (**B**) stress-strain response for the compressive-to-tensile longitudinal loading; (**C**) chains breakage at a longitudinal tensile loading above the maximum load on the above stress-strain graph


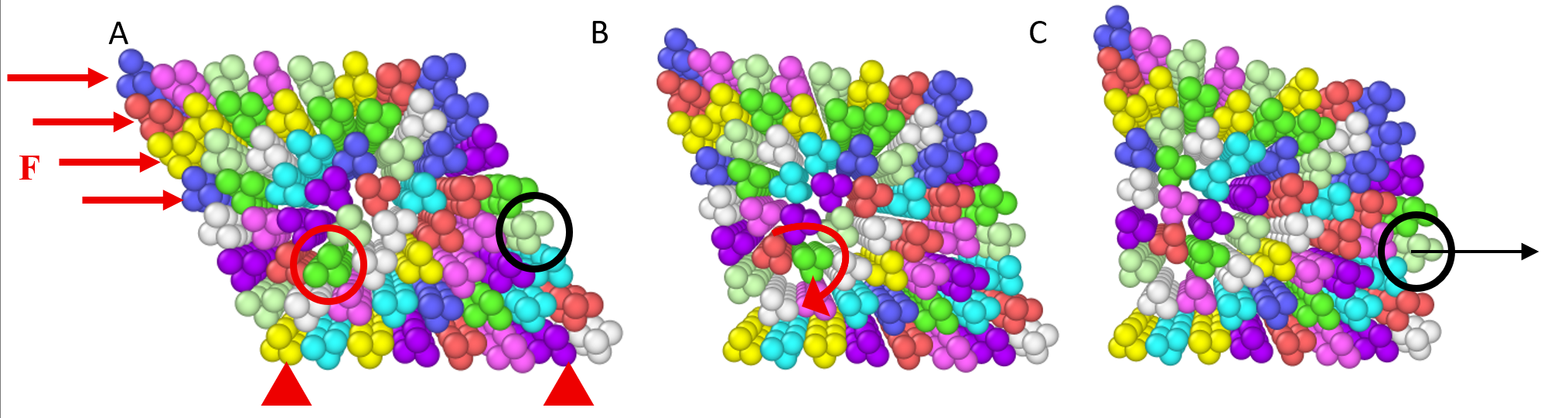


**Fig.** **S15** (**A**) Transversal loading setup for the second loading case where the top atoms are loaded on the chains transversal direction in shear, and the bottom atoms are fixed in place; (**B**) chains twisting mechanism at the imminence of chains gliding. **C** chains gliding along the gliding plane (0 1 0). The red circle highlights the Te chain where twisting can be observed through shear loading. The black circle and the black arrow highlight the Te chain where chain gliding is observed as well as the gliding direction.


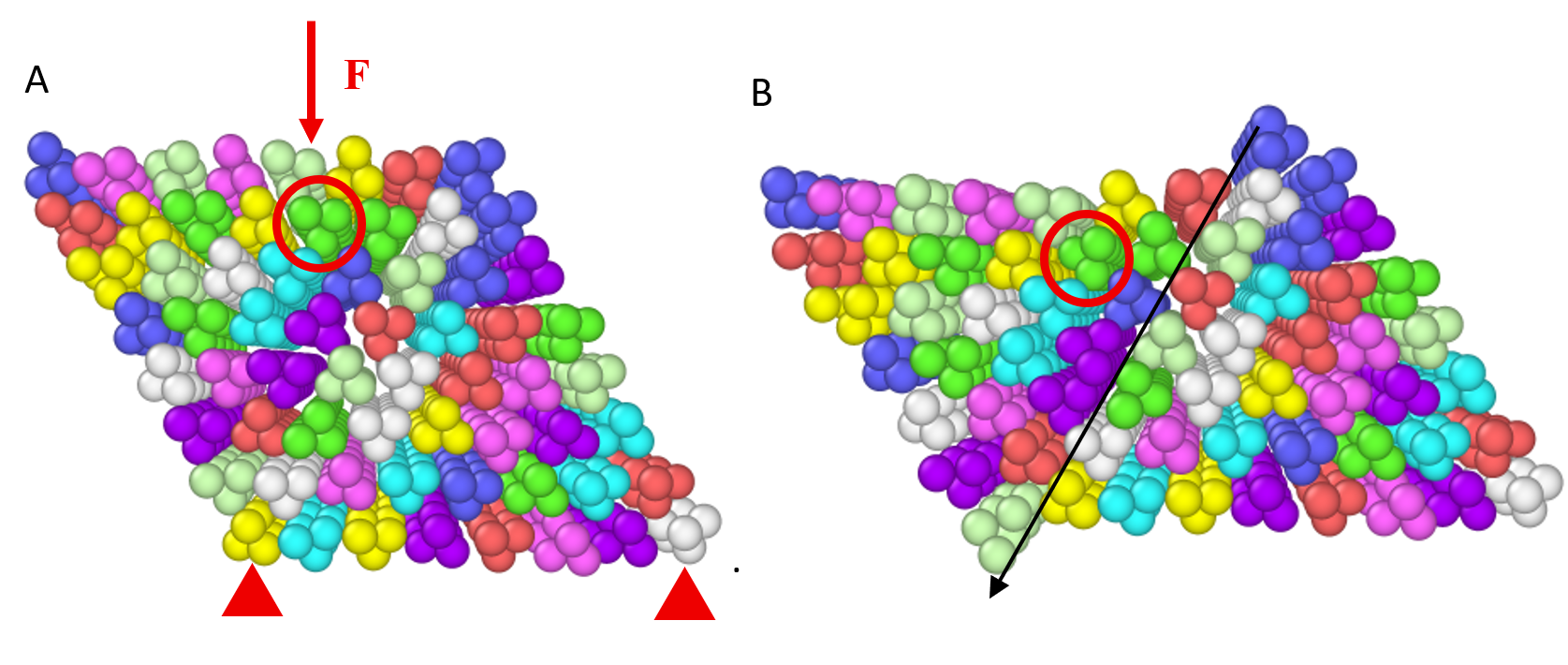


**Fig.** **16** (**A**) Transversal loading setup for the third loading case where the top atoms are loaded on the chains transversal direction in compression, and the bottom atoms are fixed in place; (**B**) chains twisting and gliding mechanisms for the third loading cas

**Table S1** Interatomic interactions models and coefficients used in the MD simulations

| System configuration | Interaction type | Interaction model | Coefficient | Value | Unit |
| --- | --- | --- | --- | --- | --- |
| Intrachain interactions | 2-body | Lennard-Jones | 𝜎_𝑇𝑒/𝑇𝑒_ | 2.4823 | Å |
|  |  |  | 𝜀_𝑇𝑒/𝑇𝑒_ | 2.6 | eV |
|  | 3-body | Harmonic angular | 𝐶_𝛼_ | 16.0 | eV |
|  |  |  | 𝜃_0_ | 98.437 | degrees |
|  | 4-body | Harmonic dihedral | 𝐶_𝐷1_ | 0.6 | eV |
|  |  |  | 𝐶_𝐷2_ | 19 | --- |
| Interchain interactions | 2-body | Lennard-Jones | 𝜎_𝑇e1/𝑇𝑒2_ | 3.36 | Å |
|  |  |  | 𝜀_𝑇𝑒1/𝑇𝑒2_ | 0.04 | eV |
| Chain-substrate interaction | 2-body | Lennard-Jones | 𝜎_𝑇𝑒/Si_ | 3.0 | Å |
|  |  |  | 𝜀_𝑇𝑒/Si_ | 0.5 | eV |
|  |  |  | 𝜎_𝑇𝑒/O_ | 2.0 | Å |
|  |  |  | 𝜀_𝑇𝑒/O_ | 0.05 | eV |
